# A mathematical model of cell-free transcription-translation with plasmid crosstalk

**DOI:** 10.1093/synbio/ysaf011

**Published:** 2025-06-14

**Authors:** Yue Han, Alexandra T Patterson, Fernanda Piorino, Mark P Styczynski

**Affiliations:** School of Chemical and Biomolecular Engineering, Georgia Institute of Technology, Atlanta, GA 30332, United States; School of Chemical and Biomolecular Engineering, Georgia Institute of Technology, Atlanta, GA 30332, United States; School of Chemical and Biomolecular Engineering, Georgia Institute of Technology, Atlanta, GA 30332, United States; Cellular Engineering Group, National Institute of Standards and Technology, Gaithersburg, MD 20899, United States; School of Chemical and Biomolecular Engineering, Georgia Institute of Technology, Atlanta, GA 30332, United States

**Keywords:** Cell-free systems, gene expression, mathematical modelling, synthetic biology, systems biology

## Abstract

Cell-free expression (CFE) systems are emerging as a powerful tool in synthetic biology, with diverse applications from prototyping genetic circuits to serving as a platform for point-of-care biosensors. When multiple genes need to be expressed in the same CFE reaction, their DNA templates (often added as plasmids) are generally assumed to behave independently of each other, with neither affecting the other’s expression. However, recent work in *Escherichia coli* CFE systems shows that multiple aspects of these templates can lead to antagonistic or synergistic interactions in expression levels of individual genes, a phenomenon referred to as plasmid crosstalk. Plasmid crosstalk can confound efforts for precise engineering of genetic circuits and even give rise to misleading observations about circuit function. Unfortunately, current mathematical and computational models are incapable of reproducing critical aspects of plasmid crosstalk. To address this gap, we created an ordinary differential equation model incorporating mechanisms to account for competition for transcription, translation, and degradation resources, as well as toxic molecule build-up. This model can recapitulate the predominant observed phenomena of plasmid crosstalk. Simulation results and subsequent experimental validation provided insights into the different sources of burden and interactions in CFE systems, including that translation is negatively impacted by macromolecular crowding caused by possibly both transcription and translation. This model thus enables deeper understanding of CFE systems and could serve as a useful tool for future CFE application design.

## Graphical Abstract

**Graphical Abstract:**
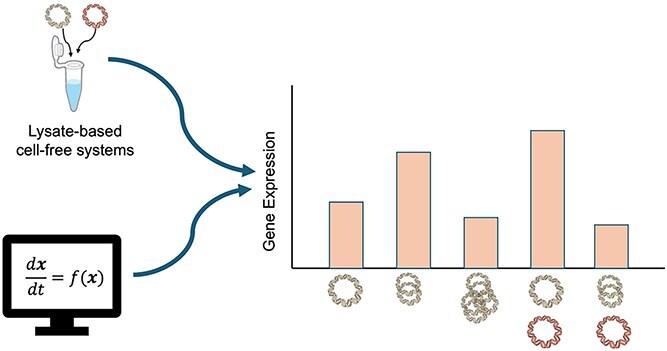

## Introduction

Cell-free expression (CFE), or cell-free protein synthesis, uses cellular transcription and translation machinery in an *in vitro* environment for the production of RNA or proteins [1]. Since their inception almost a century ago, CFE systems have been instrumental in advancing our understanding of complex biological processes [2], enabling on-demand protein manufacturing [3], point-of-care diagnostics [4–6], prototyping metabolic pathways [7, 8], and serving as a platform for rapid Design-Build-Test-Learn cycles [9–11]. CFE systems offer several unique advantages over the use of whole cells, including the ability to produce proteins difficult to synthesize in cells due to gene regulation or toxicity and the potential for bypassing the need for cell transformation in synthetic biology workflows. Advances over the past 25 years have improved yields, lowered costs, and expanded production scales, making CFE a promising tool for diverse applications [12].

*Escherichia coli*-based CFE systems remain the most commonly used and studied, although there have been significant advancements in CFE for other microorganisms [13–16]. There are two broad classes of *E. coli* CFE systems: crude lysate-based systems where the genome and cell membrane are removed [17–19] and reconstituted systems using purified components, like the PURE system [20–23]. While reconstituted systems are more simplified and often easier to predict the behaviour of since they include only the necessary enzymes and reagents in their optimal concentrations, their high cost limits their use in large-scale applications. In addition, crude lysates usually have a higher protein yield and allow more user tuning and control compared to reconstituted systems. Consequently, crude lysate-based systems have been used widely in the field due to their affordability and are the focus of this work [24].

When multiple genes need to be expressed in the same CFE reaction, their DNA templates (often added as plasmids) are generally assumed to behave independently of each other, with neither affecting the other’s expression. However, recent experimental studies have shown that the expression of one gene can often be affected by the addition of other DNA templates even when they do not share regulatory interactions, a phenomenon referred to as plasmid crosstalk [25, 26]. (DNA templates will hereafter be referred to as plasmids, even though the same phenomena can also be observed if linear DNA templates are used.) Depending on regulatory elements and plasmid concentrations, plasmid crosstalk could lead to an increase (positive crosstalk) or a decrease (negative crosstalk) in the expression of one gene upon the addition of another plasmid. These phenomena have been attributed to competition for transcription-translation (TXTL) machinery and other resources for negative crosstalk [26, 27] and competition for ribonucleases for positive crosstalk [25, 26]. These phenomena occur somewhat independently, yielding a complex interplay of positive and negative crosstalk leading to often difficult-to-predict results. Moreover, quantitative levels of plasmid crosstalk are expected to vary based on the lysate batch used as the basis for any given CFE reaction. Taken together, it is currently challenging to predict the extent and direction of plasmid crosstalk. Therefore, there is a critical need to develop a mathematical model to illustrate and predict plasmid crosstalk.

Quantitative models describing TXTL processes in CFE systems have previously been developed to investigate resource utilization and predict protein expression [28]. Ordinary differential equation (ODE)-based kinetic models have been the most widely used modelling approach for CFE, including for reconstituted systems [29–32]. For crude lysate-based CFE systems, Karzbrun *et al.* first developed a coarse-grained ODE model describing transcription and translation as single-step reactions for protein synthesis and describing mRNA and protein degradation as single-step zeroth-order reactions [33]. Niess *et al.* used a fine-grained TXTL model including the binding of multiple initiation factors and elongation factors to identify the kinetic bottleneck in cell-free translation [34]. A biophysical model developed by Marshall and Noireaux captures the TXTL dynamics of the endogenous sigma factor 70 and competition for RNA polymerases and ribosomes [35], and one developed by Adhikari *et al.* captured the dynamics of two synthetic genetic circuits [36]. However, both models assume that resources such as nucleoside triphosphates (NTP) and amino acids are infinite. To account for resource limitations in the CFE system, Moore *et al.* formulated a model explicitly tracking their concentrations and applied it to non-model organism-based CFE systems [27]. The Murray group has developed multiple models and toolboxes to account for competition for RNA polymerase, NTPs, and amino acids for the entire duration of cell-free reactions [37–39].

However, at least two reliably observed experimental crosstalk trends cannot yet be captured by existing models. First, there is typically a decrease in protein expression as plasmids containing genes with relatively strong promoters are added at high concentrations. While mathematical formulations representing resource competition can simulate a saturation of expression levels after a certain plasmid concentration due to the depletion of a limiting resource, the decrease in expression cannot be captured. Second, since mRNA degradation is often modelled with first-order kinetics, competition for mRNA degradation machinery cannot be fully captured to reproduce positive crosstalk. Beyond these two trends, there still remains much more to be characterized, explained, and predicted about lysate-based CFE systems, including variability among lysate batches [40], the tradeoff between transcription and translation [41], and the cause of their limited lifespan [1].

In this work, we developed and incorporated mathematical formulations into an ODE model of TXTL to account for key phenomena, including potential toxic molecule build-up and competition for mRNA degradation machinery, allowing the model to address critical gaps in current prediction and understanding of CFE systems. After refinement of different potential formulations and investigation of parameter space using surrogate objective function terms to select for parameterizations exhibiting desired qualitative behaviours, we showed that the model can capture key experimental trends of plasmid crosstalk and can suggest some of the factors that lead to the stalling of protein synthesis. We experimentally tested hypotheses generated from this model, finding that macromolecular crowding caused by a combination of mRNA and protein can lead to the halt of translation. This is the first CFE model capable of capturing positive plasmid crosstalk, giving it significant potential utility in applications including pathway and genetic part prototyping.

## Materials and methods

### Dataset description and processing

Experimental datasets used for fitting the plasmid crosstalk model are available in [25] with a more detailed description; they are also reproduced in Supplementary Figs S1 and S2. Briefly, the datasets consist of time-course fluorescence values for both mRNA-level crosstalk and protein-level crosstalk. The reporter used to study mRNA-level crosstalk is a 3-Way Junction dimeric-Broccoli (3WJdB) RNA aptamer, which upon transcription binds to a dye molecule present in excess in the reaction to produce a fluorescent signal. The reporter used to study protein-level crosstalk is superfolder green fluorescent protein (sfGFP). Expression of the two reporters is controlled by promoters with varying strengths: (i) a strong T7 promoter (‘T7 strong’), (ii) a weak T7 promoter (‘T7 weak’), (iii) a strong σ^70^ promoter (‘σ^70^ strong’), and (iv) a weak σ^70^ promoter (‘σ^70^ weak’). The 3WJdB RNA aptamer with σ^70^ weak gives too low of a signal to be distinguishable from noise and was thus excluded. Four different combinations of plasmids are available in the datasets: (i) reporter plasmid only, (ii) reporter plasmid with an empty vector that has no promoter to drive expression, (iii) reporter plasmid with an empty vector that has no gene insert but a T7 strong promoter, and (iv) reporter plasmid with an empty vector that has no gene insert but a σ^70^ strong promoter. All plasmids carry a kanamycin resistance gene (*kanR*) that is also expressed. Data for each plasmid combination is available for each reporter promoter strength at seven initial reporter plasmid concentrations. The fluorescence values from the original work were converted from arbitrary units to concentrations using calibration curves whose generation is described in Figs S3 and S4.

### Software toolbox and model construction

The txtlsim toolbox [38] developed in MATLAB SimBiology was used for model development. Information on lengths of promoters, genes, ribosome binding sites, and untranslated regions was provided to the toolbox for model construction. The 3WJdB RNA aptamer and empty vector were treated as untranslated regions in the plasmid. All genes capable of transcription (including antibiotic resistance markers present on each plasmid) are treated as individual ‘plasmids’ for model construction, for a total of 10 ‘plasmids’ added to the model (sfGFP, 3WJdB RNA aptamer, and empty vectors on 4, 3, and 2 promoters with different strengths, respectively, plus the *kanR*). The TXTL reactions were then constructed and added to the model object using the txtlsim toolbox (see Supplementary data ‘Model Reaction Network’). Different combinations of plasmids and concentrations were simulated using the *ScheduleDose* object in the SimBiology toolbox, where plasmids added to the lysate-based CFE systems are treated as bolus doses. Using this analogy to dose response, 112 sets of time-course data (4 promoters for reporter gene, 7 initial concentrations, and 4 combinations of plasmids) were calculated for sfGFP expression, and 84 sets of time-course data (3 promoters for reporter gene instead of 4) for 3WJdB RNA aptamer. The built ODEs were solved using SUNDIALS [42].

### Parameter sampling

Parameters were sampled in the bounds in Supplementary Table S1 to generate models (see supplementary ‘Model Parameter Bounds’). To sample the high-dimensional space effectively, Latin hypercube sampling (LHS) [43] was used to generate 1 000 000 sets of parameters within the bounds, but based on the logarithm of the parameter values. Heuristics based on biological insights were then used to filter out some of these parameters. Specifically, we applied the following constraints: (i) the binding affinity for T7 RNA polymerase of T7 strong is stronger than that of T7 weak; (ii) the binding affinity of σ^70^ weak for native RNA polymerase is weaker than that of σ^70^ strong for native RNA polymerase and that of T7 weak for T7 RNAP; (iii) the binding affinity of sfGFP and 3WJdB aptamer mRNA to RNA ribonucleases is weaker than that of *kanR* mRNA and the empty vector due to the presence of stabilizing hairpin secondary structures in the UTRs of the reporter genes; and (iv) the degradation kinetic constant is lower for the kanamycin resistance, sfGFP, and 3WJdB mRNA than for the empty vector due to their longer length. The constraints cut the number of sampled parameter sets to 159 044 for further analysis.

### Parameter estimation

Parameters were estimated for alternative toxic molecule build-up mechanisms and for RNA-level crosstalk using the ‘fit’ function in the SimBiology toolbox with 48 and 96 initial guesses, respectively. The initial guesses were sampled from a uniform distribution between the upper and lower bounds in Supplementary Table S1 but based on the logarithm of the parameter values. The ‘fit’ function uses ‘lsqnonlin’, which implements the Levenberg–Marquardt algorithm to perform a maximum likelihood estimation for the average of 3 replicates of the experimental data.

### Surrogate terms for sfGFP qualitative experimental trends

**Figure 1:**
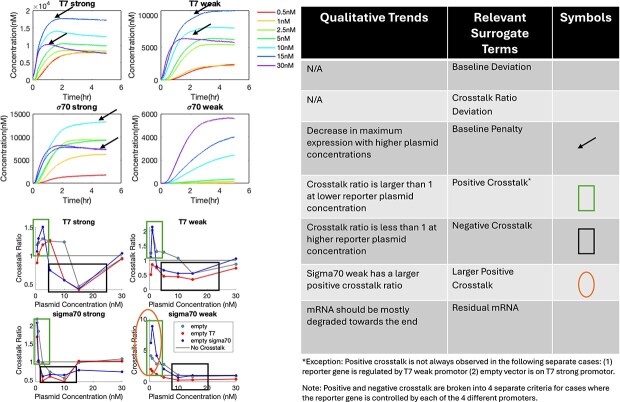
Surrogate terms visualized in the context of experimental data. Error bars in plots are removed for the sake of clarity.

To screen for desired experimental trends effectively, we devised surrogate terms to mathematically represent the desired qualitative trends of experimental data (Fig. 1). This approach drew its inspiration from the concept of surrogate models [44], which are simplified approximations of more complex models that capture the input–output relationship, and reinforcement learning [45], where reward terms, often qualitative, are used to train models toward desired behaviours. (We acknowledge that typical motivations and applications for surrogate models and reinforcement learning are different from this application.) These surrogate terms are calculated based on the 112 simulated time-course sfGFP expression datasets, and were all defined such that a more negative value is preferable.

Crosstalk ratios are calculated using the sfGFP concentration at the 3-h timepoint as in the experimental data based on expression levels with and without an empty plasmid. They are then used to calculate the crosstalk ratio deviation term, positive and negative crosstalk terms, and the large positive crosstalk term.

The baseline penalty is calculated as a sign indicator (−1 or 1) times the logarithm of the absolute difference between the terminal sfGFP concentration at 30 nM of reporter plasmid and that at a lower reporter plasmid concentration. For reporters under the control of strong promoters, the sign indicator is chosen as positive if the 30 nM condition yields the highest fluorescence value and negative otherwise. For the σ^70^ weak promoter, the sign indicator is chosen as negative if the 30 nM condition yields the highest fluorescence value and positive otherwise.

Since positive crosstalk is experimentally observed at only lower reporter plasmid concentrations, the positive crosstalk term is defined to be 1 (no crosstalk) minus the maximum crosstalk ratio among 0.5, 1, and 2.5 nM reporter plasmid concentrations. Similarly, since negative crosstalk is experimentally observed at higher reporter plasmid concentrations, the negative crosstalk term is defined to be the minimum crosstalk ratio among 5, 10, 15, and 30 nM reporter plasmid concentrations minus 1. A graphical illustration of these surrogate terms with experimental data is given in Fig. 1.

The goals (Supplementary Table S2) are threshold values that, when achieved, yield models that reasonably capture the qualitative experimental trends. For the deviation-related and residual mRNA surrogate terms, since there is no hard constraint on their values, the median of their values calculated from all the sampled parameter sets was heuristically used as the goal. This *de facto* means that the lower 50% of parameter sets were eliminated.

For multiple surrogate terms, the theoretical threshold value to enforce the presence of a phenomenon would be zero; however, small-magnitude values numerically lower than the zero threshold often did not qualitatively and satisfactorily capture the desired experimental trends. Based on preliminary tuning investigations, the baseline penalty goal was set to be −1e−04, and goals for penalties for the crosstalk ratio were set to be −0.05 to ensure that the trends were more than just trivially satisfied.

### Parameter estimation with surrogate terms

Two modes of optimization were performed with the surrogate terms: parameter estimation mode and feasibility mode. In parameter estimation mode, surrogate terms were scaled such that they all fall between −15 and 15, and the sum of these scaled surrogate terms was used as the objective function to be minimized. In this case, these terms are treated as soft constraints. The fmincon solver was used for optimization, and parameter bounds described in Supplementary Table S1 were enforced. Considering the high computational cost of the optimization, an initial 10 000 LHS samples were used, and 1591 filtered initial guesses were used. In feasibility mode, the surrogate terms were also treated as hard constraints rather than soft constraints, but the parameter bounds were eliminated to increase the feasible parameter space. One hundred initial points were sampled from a uniform distribution between 1e−08 and 1e+08, but based on the logarithm of the parameter values. The fmincon solver in MATLAB was used for optimization with the feasibility mode, which minimizes the infeasibility instead of directly attempting to optimize for the objective.

### *In vitro* transcription

RNA coding for sfGFP with and without UTR, RBS, and start codon was transcribed from linear DNA templates (Supplementary Table S4) using the HiScribe T7 High Yield RNA Synthetics kit according to the manufacturer’s protocol (New England Biolabs). Following RNA synthesis, DNase I (Zymo Research) was added to degrade the linear DNA template. The RNA products were then purified using an RNA Clean and Concentrator kit (Zymo Research) according to the manufacturer’s protocol. Following purification, RNA concentration was measured on a NanoDrop 2000, sub-aliquoted to reduce freeze–thaw cycles, and stored at −80 °C.

### Lysate preparation

Cellular lysate was prepared as previously described [25]. BL21 Star (DE3) *ΔlacZ* or BL21 (DE3) *ΔlacZ* cells were grown in 2× YTP medium at 37°C and 180 rpm to an OD of 1.7, which corresponded with the mid-exponential growth phase. Expression of T7 RNA polymerase was induced with 0.4 mM isopropyl β-d-1-thiogalactopyranoside around OD 0.6. Cells were then centrifuged at 2700 rcf and washed three times with S30A buffer, which contains 50 mM tris, 14 mM magnesium glutamate, 60 mM potassium glutamate, and 2 mM dithiothreitol, and is pH-corrected to 7.7 with acetic acid. After the final centrifugation, the wet cell mass was determined, and cells were resuspended in 1 ml of S30A buffer per 1 g of wet cell mass. The cellular resuspension was divided into 1 ml aliquots. Cells were lysed using a Q125 Sonicator (Qsonica, Newton, CT) at a frequency of 20 kHz and 50% of amplitude. Cells were sonicated on ice with three cycles of 10 s on and 10 s off, delivering ~300 J, at which point the cells appeared visibly lysed. An additional 4 mM of dithiothreitol was added to each tube, and the sonicated mixture was then centrifuged at 12000 rcf and 4°C for 10 min. The supernatant was removed, divided into 1 ml aliquots, and incubated at 37°C and 220 rpm for 80 min. After this runoff reaction, the cellular lysate was centrifuged at 12000 rcf and 4°C for 10 min. The supernatant was removed and loaded into a 10 kDa MWCO dialysis cassette (Thermo Fisher). Lysate was dialyzed in 1 L of S30B buffer (14 mM magnesium glutamate, 60 mM potassium glutamate, 1 mM dithiothreitol, pH-corrected to 8.2 with Tris) at 4°C for 3 h. Dialyzed lysate was removed and centrifuged at 12000 rcf and 4°C for 10 min. The supernatant was removed, aliquoted, and stored at −80°C for future use.

### Cell-free reactions

Cell-free reactions were run as previously described [25]. Each cell-free reaction contained 0.85 mM each of GTP, UTP, and CTP, in addition to 1.2 mM ATP, 34 μg/ml folinic acid, and 170 μg/ml *E. coli* tRNA mixture, 130 mM potassium glutamate, 10 mM ammonium glutamate, 12 mM magnesium glutamate, 2 mM each of the 20 standard amino acids, 0.33 mM nicotine adenine dinucleotide, 0.27 mM coenzyme-A, 1.5 mM spermidine, 1 mM putrescine, 4 mM sodium oxalate, 33 mM phosphoenolpyruvate (PEP), 27% cell extract, and RNA concentrations specified for each experiment.

Reactions were run in 10 μl volumes in 384-well small-volume plates (Greiner Bio-One), and a clear adhesive film was used to cover the plate and prevent evaporation. Plates were incubated for 3 h at 37°C in a plate reader (Synergy4, BioTek), and fluorescence was measured every 5 min. Excitation and emission wavelengths for sfGFP were 485 and 510 nm, respectively. Excitation and emission wavelengths for 3WJdB were 472 and 507 nm, respectively.

## Results

### Summary of evidence for plasmid crosstalk

**Figure 2:**
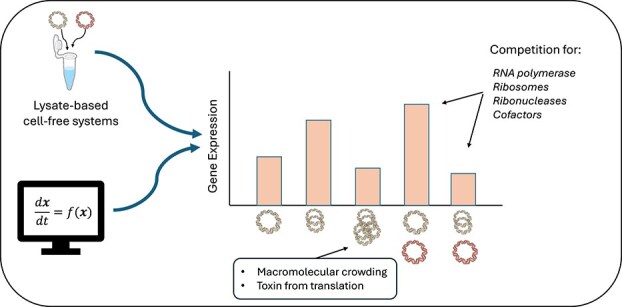
Illustration of CFE phenomena, including plasmid crosstalk, and hypotheses about their underlying causes. Cell-free expression increases with increasing plasmid concentration in low concentration regimes, but as the concentration of plasmid gets very high, total expression decreases; this phenomenon is hypothesized to be due to macromolecular crowding or toxins produced from translation. When multiple different plasmids are added, they compete for various resources (e.g. polymerases or nucleases), causing the expression to be higher or lower than the baseline depending on whether the resources lead to production or degradation of the gene product. These hypotheses were integrated into a computational model.

In previous experimental findings, expression of a reporter protein (sfGFP) and a reporter RNA aptamer (3WJdB) at varying initial plasmid concentrations were studied under co-expression of 10 nM of additional plasmid with a kanamycin resistance cassette but no coding sequences. Plasmid crosstalk was observed on both the mRNA and protein levels, and hypotheses were posited about the root causes of the different types of crosstalk (Fig. 2).

On the RNA level, all 3-h time course measurements consisted of a local maximum in RNA expression followed by a decay to zero. This corresponds to the end of transcription in the system followed by degradation of mRNA due to *E. coli* ribonucleases present in the lysate. Positive crosstalk was observed in most combinations of reporter constructs and ‘empty vector’ constructs (Supplementary Fig. S2), likely due to competition for nucleases (or ‘nuclease distraction’) [25, 26].

On the protein level, positive crosstalk was generally observed at lower reporter plasmid concentrations (<2.5 nM), negative crosstalk was generally observed at moderate reporter plasmid concentrations (2.5–15 nM), and crosstalk was minimized at the highest reporter plasmid concentration (30 nM). The transition from negative to no crosstalk may be related to the overall decrease in reporter protein expression for the control (i.e. no plasmid co-expressed with reporter plasmid) observed at higher reporter plasmid concentrations. In addition, a larger positive crosstalk ratio was generally observed when the reporter gene was on a weaker promoter (Fig. 1).

It has been previously shown that existing models are not able to capture positive crosstalk [25]. Since the current hypothesis for positive crosstalk is of competition for RNA degradation activity *via* competition for ribonucleases, models using first-order kinetics for RNA degradation will not be able to capture this behaviour. These models implicitly assume an infinite supply of ribonucleases, since the degradation rate of one RNA is only dependent on its own concentration but not directly or indirectly on the concentrations of other RNAs. Of the existing models for transcription and translation for lysate-based CFE systems, we found that the txtlsim toolbox uses mass-action kinetics to describe RNA degradation reactions and explicitly tracks ribonucleases [38]. However, the core model as-is was not able to capture observed positive crosstalk trends (Supplementary Fig. S5).

Previously reported data also exhibited baseline reporter gene expression (without empty vectors or crosstalk) decreases at higher plasmid concentrations (Fig. 1). This phenomenon also could not be reproduced with existing models of cell-free metabolism; these models only capture depletion of resources, and thus the expression only saturates with plasmid dosage and never decreases. The exact mechanism underlying this observed phenomenon remains uncertain, although the build-up of toxic metabolites has been hypothesized as one potential cause [25, 46, 47].

### Model modification

To capture the two key experimental trends—decrease in expression at higher plasmid concentrations and increase in expression with additional plasmids (positive crosstalk)—two changes were made to the structure of the model.

First, we added a mechanism for toxic molecule generation in lysate-based CFE to capture the decrease in protein expression at higher plasmid concentrations. We noticed that in protein-level crosstalk, a higher concentration of plasmid is generally correlated with similar initial translation rates but early termination of gene expression, ultimately resulting in a decrease in total protein expression. Previous cell-free work that successfully extended the lifespan of lysate-based CFE systems has used strategies ranging from supplementing small molecules to continuous reaction using dialysis [48, 49]. Accumulation of inorganic phosphate due to the use of phosphorylated energy substrates such as PEP is hypothesized to have a negative impact on protein expression due to its binding to magnesium ions, which are essential for protein expression [48, 50, 51]. Alternative energy sources for central carbon metabolism have been explored to decrease the accumulation of inorganic phosphate in hopes of improving protein expression [41, 52–55], but limitations on success in these efforts point to likely interplay with various other molecules like acetate, lactate, and cofactor balances. More recently, we have also identified endogenous metabolism in lysate-based CFE systems as having a substantial impact on protein expression level [56, 57]. These studies collectively point to the impact of small molecules on the TXTL lifespan of lysate-based CFE systems, although the exact identities of the small molecules with important impacts remain unknown. Therefore, we hypothesized that the negative crosstalk seen in the experimental work may be due to the build-up of some unknown, toxic molecule whose levels can be correlated with protein expression. To model this hypothesis, we proposed a toxic molecule build-up mechanism with the following characteristics: (i) toxic molecules are generated during TXTL, (ii) lysate-based CFE has the ability to buffer these toxic molecules to some extent, as evidenced by the fact that decreasing expression only happens at extremely high plasmid concentrations, and (iii) past a certain threshold, the toxic molecules poison TXTL. We added ‘toxin’ as a state variable and devised the following mechanism:


$$ \frac{d\left[ toxin\right]}{dt}=\sum{v}_{tl}-b $$

$$ {r}_{Ribo,\mathit{\deg}}={k}_{toxin}\bullet \left(1+\tanh \left(\left[ toxin\right]-{\left[ toxin\right]}_{\mathrm{threshold}}\right)\right)\bullet \left[ Ribo\right] $$

where $\sum{v}_{tl}$ is the sum of translation rates, $b$ is a constant representing the buffering capacity of the lysate-based CFE, ${k}_{toxin}$ is the toxin coefficient representing the impact of toxic metabolite build-up on translation, and ${\left[ toxin\right]}_{threshold}$ represents the threshold toxin concentration above which translation is compromised *via* degradation of ribosome. The hyperbolic tangent term allows for a near-step change increase in toxin impact at the threshold concentration while still keeping the function continuously differentiable for the computational ODE integrator. We note that the toxin state variable here does not refer to any specific metabolite, but instead mathematically indicates the status of the CFE system. This specific mechanism was found to be most effective in capturing the decrease in expression at high plasmid concentrations compared to other tested mechanisms (see section ‘Translation is negatively impacted by mRNA-induced crowding’ below).

Second, to capture the positive crosstalk that is hypothesized to be caused by the competition of mRNA for ribonucleases, we found that we needed to make the following modifications: (i) the initial concentration of ribonuclease was assumed to be much lower than the original 20 mM used in the txltsim toolbox; (ii) reporter mRNAs were assumed to have a lower binding affinity to ribonucleases than the *kanR* and empty vector RNA due to the presence of a stabilizing hairpin in the UTR and a stemloop secondary structure in sfGFP & 3WJdB mRNAs, respectively; and (iii) the reporter mRNAs and the *kanR* mRNA were assumed to have a lower degradation rate constant than any empty vector RNAs since they are significantly longer than the empty vector RNAs.

We also hypothesized that after initial (partial) degradation by ribonuclease, the resulting non-functional mRNA may still occupy the RNA degradation machinery, as other binding sites may still be available even after an endoribonuclease cleavage. Two alternative mRNA degradation mechanisms were proposed and studied in the context of mRNA degradation data, and the proposed mechanisms were able to explain experimental observations (Supplementary Figs S6 and S7). However, the parameters were not identifiable even for the simpler mechanism, indicating the potential for overfitting, and so we decided to move forward with mass-action kinetics that allowed for similar fitting accuracy.

### The modified TXTL model can capture key experimental trends

**Figure 3:**
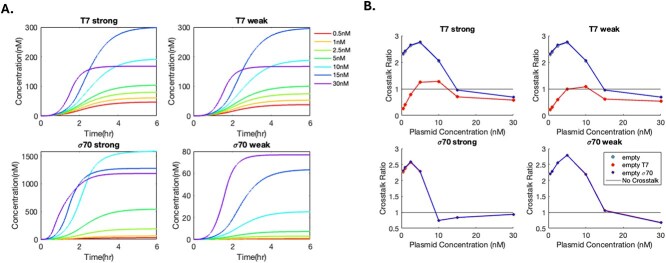
Simulation with selected model parameterization. (A) sfGFP expression time-course for the baseline case (reporter plasmid only) at seven reporter plasmid concentrations and four promoter strengths. (B) Crosstalk ratios for sfGFP expression upon addition of empty plasmid vectors as described in the legend. Values larger and smaller than one indicate positive crosstalk and negative crosstalk, respectively.

After initial attempts at parameter estimation, we decided that there are inherent challenges due to model complexity, measurement noise, and differences between experimental conditions such that using a typical objective function to minimize sum of squared error is insufficient to identify parameters that allow the model to capture key qualitative phenomena (Supplementary Figs S8 and S9). As a result, we prioritized capturing qualitative experimental trends over absolute quantitative expression values and adopted a parameter sampling approach to explore the model’s behaviour. In total, 159 044 sets of sampled parameters satisfying parameter heuristics were used to generate time-course data used as the basis for calculating surrogate terms representing key qualitative trends. These surrogate terms were then categorized into nine criteria (Supplementary Table S2), and the number of criteria satisfied was evaluated for each sampled parameter set. No model parameterization was found to satisfy all 9 criteria (Supplementary Table S3). In total, 16 parameterizations were found to satisfy 8 out of the 9 criteria, and only 1 of these satisfied all criteria for the two key experimental trends, baseline penalty and positive and negative crosstalk (shown as T7 strong, T7 weak, σ^70^ strong, and σ^70^ weak crosstalk) (Supplementary Fig S10). The reporter-only expression profiles and the crosstalk ratio profiles for this model are shown in Fig. 3.

While there is a discrepancy in the simulated protein concentrations compared to the absolute experimental values shown in Fig. 1, the critical experimental trends in plasmid crosstalk are captured. First, the decrease in total protein expression at 30 nM compared to expression at 15 nM is captured in T7 strong, T7 weak, and σ^70^ strong cases, while in the σ^70^ weak case, the toxin mechanism is not triggered at 30 nM initial plasmid concentration, in line with experimental data (Fig. 3A). The transition from positive to negative crosstalk with increasing plasmid concentrations is also captured, as indicated by the transition of crosstalk ratios from greater than 1 to less than 1 (Fig. 3B). However, the crosstalk ratios for the empty vector case (grey) and that for the empty vector with a σ^70^ strong promoter (blue) are predicted to be the same, whereas experimentally the σ^70^ strong empty vector has differing crosstalk ratios at the same reporter plasmid concentration. This could be due to the fast degradation of the empty vector and a lack of resource competition predicted by the model, leading to an underestimation of TXTL burden caused by the empty vectors, or could be due to some unknown mechanism not included in the model.

In addition, the initial spike in crosstalk ratios at low reporter plasmid concentrations before decreasing again was also captured by the model even though it was not explicitly added as a separate surrogate term. Near-neutral crosstalk at higher reporter plasmid concentrations was also captured by the model. However, the experimental trend that the positive crosstalk ratio should be significantly higher for the σ^70^ weak case than in other cases is not well captured in this model.

The selected model is also able to capture the positive crosstalk observed at the mRNA level (Supplementary Fig S11), although there is again a discrepancy in predicted absolute mRNA concentrations due to the absence of direct model calibration to experimental data. With direct training with experimental data, the model structure is able to predict mRNA level crosstalk (Supplementary Fig S2).

### Model provides insight into possible causes of plasmid crosstalk

Although the selected model is not able to capture all qualitative experimental trends, it does allow some insights into the possible causes of plasmid crosstalk by tracking the unobservable reaction intermediates in CFE systems.

**Figure 4:**
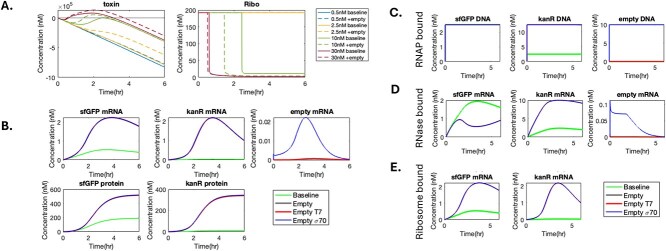
Resource utilization of the selected model. (A) Time-course data for toxin and ribosome concentrations for cases with different sfGFP reporter plasmid concentrations, with and without the additional plasmid. Toxin produced by the additional plasmid plays a more important role at 10 nM in terminating translation, compared to 0.5 nM and 30 nM. (B) The concentration profile of mRNA (excluding those bound to ribonucleases) produced for sfGFP, *kanR*, and empty genes and of KanR and sfGFP protein over the simulated course of 6 h for 2.5 nM of reporter plasmid. (C) Concentration of RNA polymerase bound with each of the sfGFP, *kanR*, and empty vector gene over time. (D) Concentration of ribonucleases bound with each of sfGFP, *kanR*, and empty mRNA over time. (E) Concentration of ribosomes bound with sfGFP and *kanR* mRNA (excluding those bound to ribonucleases) over time.

Looking at the toxin level at low and high plasmid concentrations, the toxin mechanism is only activated for an extended period of time at high plasmid concentrations (Fig. 4A). (We note that while the toxin level goes to unrealistic negative values since it is not biologically relevant, it did not prevent the mechanism from accurately reproducing the dependence of total expression on plasmid concentration.) The activation of the toxin mechanism leads to a significant decrease in ribosome concentrations, which corresponds to the early end of translation also observed in experimental data. The observed experimental trend that negative crosstalk is most prominent at 10 or 15 nM instead of 30 nM reporter plasmid concentration can be explained by this mechanism: at 30 nM, the baseline expression alone triggers the early termination of translation (solid red line), and the additional expression with the empty plasmid has a marginal effect (dashed red line); at 10 nM, the baseline expression alone does not trigger termination until ~2.5 h (solid green line), but the additional expression significantly accelerates the termination (dashed green line) and causes an overall lower expression level.

Next, we analysed the concentrations of several unobservable reaction intermediates over time for two experimental conditions to explore the possible causes of positive crosstalk and negative crosstalk. The 2.5 and 10 nM (Supplementary Fig S12) reporter plasmid concentrations in σ^70^ strong cases were used as a case study for positive and negative crosstalk, respectively, as crosstalk is most prominent at these two concentrations in simulations. Relevant simulated concentrations in a CFE reaction containing 2.5 nM of reporter plasmid and different combinations of additional plasmids are shown in Fig. 4B–E. Positive crosstalk occurs in both mRNA and protein levels (green curve below other curves) for sfGFP (Fig. 4B).

A focus on bound species and concentrations of mRNA (Fig. 4C–E) suggests that the kanamycin resistance marker plays a significant role in the phenomenon, as it occupies more resources when its DNA is present in excess (12.5 nM) compared to the reporter DNA (2.5 nM) as shown by the higher concentrations of *kanR*-related intermediates bound to RNA polymerase, ribonucleases, and ribosomes when empty vectors are added (green curve below other curves) (Fig. 4C). For the reporter sfGFP, competition for ribonucleases between the *kanR* mRNA and sfGFP mRNA leads to a lower concentration of ribonuclease-bound sfGFP mRNA with additional plasmids (Fig. 4D), resulting in more translating sfGFP mRNA and thus more protein expression (Fig. 4E).

At a high reporter plasmid concentration of 10 nM (Supplementary Fig S12) where the ratio of reporter DNA to kanamycin resistance DNA is larger and closer to 1, the concentration of reporter mRNA bound to RNase is still higher in the baseline case than other cases with additional plasmids, indicating positive crosstalk at the mRNA level. However, the magnitude of positive crosstalk on mRNA expression is less significant compared to at lower reporter plasmid concentrations, since the ribonucleases are already close to saturation with the amount of *kanR* mRNA at this high plasmid concentration in the baseline case. This fact makes the nuclease distraction impact from the additional *kanR* on empty vectors on sfGFP mRNA less significant at this reporter plasmid concentration.

The initial ‘spike’ in crosstalk ratio at lower reporter plasmid concentrations observed in Fig. 1 and reproduced in Fig. 3B can also potentially be explained by analysis of the model. At the lowest tested 0.5 nM reporter plasmid concentration, the degradation machinery is not as completely occupied by the stronger-affinity *kanR* mRNA alone, and thus still has capacity to degrade sfGFP mRNA (Supplementary Fig S13). On the other hand, at 2.5 nM of reporter plasmid concentration (and thus with even more *kanR* mRNA), the degradation mechanism can be almost completely occupied by *kanR* mRNA alone, leaving less nuclease bandwidth for sfGFP mRNA. This phenomenon leads to a higher proportion of sfGFP mRNA shielded from ribonucleases by *kanR* mRNA in 2.5 nM than 0.5 nM, which leads to the larger positive crosstalk ratio at 2.5 nM.

Thus, using the mechanistic model we developed, we are able to look at the levels of otherwise unobservable reaction intermediates to explain some experimentally observed phenomena. We see that crosstalk is defined by a balance not only between competition for TXTL resources such as RNA polymerase, ribonucleases, ribosomes, NTPs, and amino acids, but also between increased expression and translational toxicity leading to early termination of translation in lysate-based CFE systems.

### Global parameter sensitivity analysis

**Figure 5:**
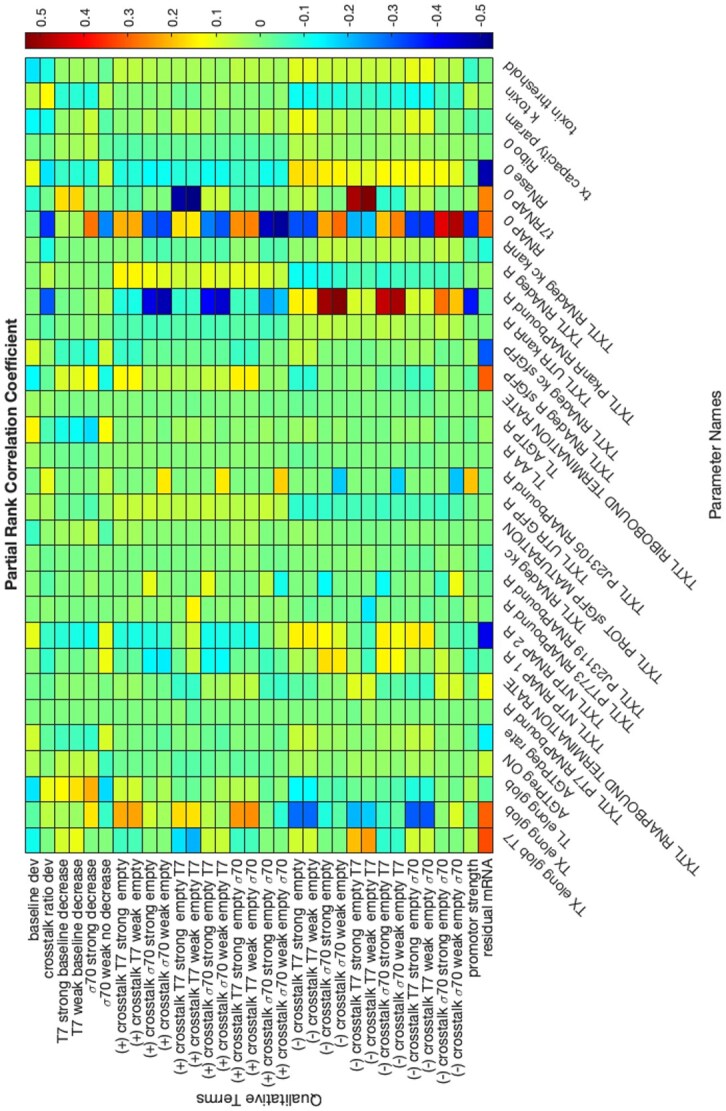
Heatmap for the partial rank correlation coefficient between surrogate terms and model parameters. Model parameters are described in Supplementary data ‘Model Reaction Network’ and Supplementary Table S1.

A correlation-based global sensitivity analysis was performed on model parameters with respect to the surrogate terms to characterize the model. Partial rank correlation coefficients were calculated for each pair of sampled surrogate term and parameter (Fig. 5). Multiple parameters were found to be correlated with several surrogate terms, which indicates that some of the experimental trends could be significantly impacted by changing the parameters within the sampled bounds; some of those correlations are discussed below. We note that, as already defined, a surrogate term that is a more negative indicates more consistency with the desired phenotypic behaviours for that respective term. Thus, a positive correlation between a surrogate term and a parameter means that a smaller value of that parameter corresponds to better reproduction of that term’s observed phenomenon.

The baseline surrogate terms for σ^70^ strong and σ^70^ weak are positively and negatively correlated with RNAP0, the initial native RNA polymerase concentration, respectively. This indicates that higher initial RNA polymerase concentration is associated with more positive, less desirable values of the σ^70^ strong baseline term (i.e. decreased protein expression at higher plasmid concentrations) but more negative, more desirable values of the σ^70^ weak baseline term (i.e. monotonic increase of protein expression with higher plasmid concentrations). This is counterintuitive at first since as the initial RNA polymerase concentration increases, there are ample resources for transcription, which should in turn lead to more mRNAs produced to potentially trigger the toxin mechanism in subsequent translation and satisfy the σ^70^ strong baseline term. One possible cause is that the toxin mechanism is activated even with a relatively low initial RNA polymerase concentration, such that the maximum effect of the toxin mechanism—with the most negative and desirable baseline surrogate term—occurs at relatively low initial RNA polymerase concentrations. As RNA polymerase levels increase, the toxin mechanism starts to affect expression at 10 nM of plasmid (i.e. decreasing protein expression at even lower reporter plasmid concentrations), while the levels at 30 nM do not change, making the difference between GFP levels at 30 nM and at 10 nM decrease. This makes the baseline term less negative, causing the apparent positive correlation between initial RNA polymerase and the σ^70^ strong baseline term.

The surrogate terms for positive crosstalk in cases with a σ^70^ regulated reporter gene are negatively correlated with the reverse binding kinetic constant of *kanR* to RNA polymerase, while the surrogate terms for negative crosstalk in cases with a σ^70^-regulated reporter gene are positively correlated with this parameter. A larger reverse binding kinetic constant indicates a weaker binding affinity. Thus, a weaker binding affinity between *kanR* and RNA polymerase is correlated with a more negative surrogate term and thus more apparent positive crosstalk, *via* the minimization of negative crosstalk. While we generally attribute much of positive crosstalk to competition for nucleases, a weaker affinity between RNA polymerase and *kanR* would serve to amplify that positive crosstalk by minimizing competition between the reporter and *kanR* for polymerases. The contribution of this affinity to the negative crosstalk surrogate term is more intuitive, as negative crosstalk is partially attributed to polymerase competition, and so a weaker affinity would necessarily decrease this competition and thus yield a less negative surrogate term for negative crosstalk.

The positive crosstalk surrogate term in cases with a T7-regulated reporter gene is positively correlated with the initial native RNA polymerase concentration, while the positive crosstalk in cases with a σ^70^-regulated reporter gene is negatively correlated with the initial RNA polymerase concentration. This trend is reversed for negative crosstalk terms. The σ^70^-regulated correlation reflects a decrease in polymerase competition with increasing initial polymerase competition along with more transcription to allow for nuclease competition, leading to positive crosstalk. On the other hand, higher initial RNA polymerase concentrations lead to more transcription and translation, triggering the toxin mechanism and potentially competition for ribosomes, thus resulting in negative crosstalk for T7-regulated reporter genes. Taken together, this supports a potential mechanism in which negative and positive crosstalk mechanisms occur simultaneously and their different magnitudes result in apparent positive or negative changes in mRNA and protein expression.

In addition, it is not surprising that the surrogate term for residual mRNA is generally positively correlated with parameters contributing to a higher transcription rate and negatively correlated with parameters contributing to a faster mRNA degradation rate.

### Translation is negatively impacted by mRNA-induced crowding

In devising the toxic metabolite build-up mechanism, multiple alternative formulations were possible. The model described above and used for the simulations in this work defined toxic build-up as a direct function of translational rates having a negative impact on translation rates, such that increased translation limits more translation. However, the toxin could in principle be generated from transcription or translation, and the toxin could have a negative impact on transcription or translation. To distinguish between these possibilities, we substituted the sum of translation rates in the above-described mechanism with the sum of transcription rates and substituted impacts on ribosome degradation with impacts on degradation of both RNA polymerases (T7 and native). All baseline expression data (no empty vectors) with a T7 strong promoter were used as a testbed for these four alternative toxin mechanisms: (i) toxin generated by transcription and decreases transcription (TX-TX); (ii) toxin generated by translation and decreases translation (TL-TL); (iii) toxin generated by translation and decreases transcription (TL-TX); and (iv) toxin generated by transcription and decreases translation (TX-TL).

**Figure 6:**
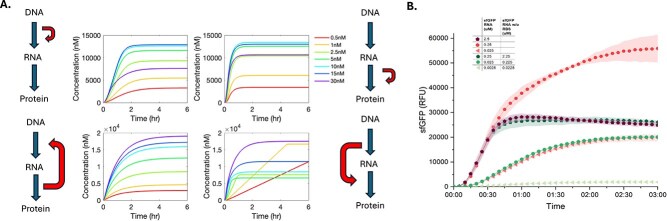
Analysis of toxic metabolite mechanism. (A) Fitting result for alternative toxic metabolite build-up models indicated next to each respective plot. The model with toxin generation by translation and negative impacts on translation reproduced the experimental result of lower total concentration for 30 nM plasmid than at some lower plasmid concentrations; the model with toxin generation by transcription and negative impacts on transcription also reproduced the same result, but is not supported by other data. (B) Experimental data on translational burden. Data points represent the average of three technical replicates, and error bars represent the standard deviation of those replicates.

The experimentally observed decrease in protein expression at higher reporter plasmid concentration was only captured by either the TX-TX or TL-TL models (Fig. 6A). However, negative crosstalk was rarely observed in the mRNA-level experimental data, suggesting that either the TX-TX model is likely not true or that at 30 nM of reporter plasmid and 10 nM of the empty vector plasmid, the threshold for adverse effects of transcription on transcription is not reached. We do note the size difference in transcripts between the mRNA reporter and protein reporter, which may contribute to some of these discrepancies. For the TX-TL case, although there may initially appear to be a decrease in protein expression at the lower concentrations and later an increase, it represents the physically irrelevant case where sfGFP maturation is the rate-limiting step in the system and yields an abrupt change of sfGFP concentration slope that is rarely observed in experimental data. This indicates that the mechanism is unlikely to reasonably explain the decreased expression at high reporter plasmid concentration. Thus, we decided to use the TL-TL model as the basis for the toxin mechanism.

The negative feedback of translation on itself was further investigated with a translation-only experimental system (Fig. 6B). For this experiment, 0.025, 0.25, and 2.5 $\mathrm{\mu}$M of *in vitro* synthesized sfGFP RNAs were added to lysate-based CFE systems to directly measure translation without transcription. There is a clear decrease in sfGFP expression from 0.25 $\mathrm{\mu}$M (dark pink) to 2.5 $\mathrm{\mu}$M (purple) of sfGFP RNA, suggesting that increased RNA concentration (and thus perhaps a higher translational rate) is leading to the loss of translational function. As an additional control, the same three total concentrations of sfGFP RNAs were added, except 90% of the RNA for each concentration have no ribosome binding sites and thus are not capable of serving as templates for translation. At 0.25 $\mathrm{\mu}$M total concentration, there is no impact on translation. However, for 0.25 $\mathrm{\mu}$M of complete reporter mRNA, the addition of non-translatable mRNA up to 2.5 $\mathrm{\mu}$M total concentration (dark green) leads to a decrease in expression almost identical to that observed at 2.5 $\mathrm{\mu}$M of complete reporter mRNA (purple). This might be attributed to decreased translational efficiency caused by RNA-induced macromolecular crowding; the direct cause of this decrease in efficiency remains unknown. Note that the initial translation rates with 0.25 $\mathrm{\mu}$M (dark pink) and 2.5 $\mathrm{\mu}$M (purple) of sfGFP RNA are the same until the early termination at 0.5 h, which is similar to what we observed in the crosstalk experimental data. We hypothesized at least two potential causes of this early termination: (i) there is a threshold over which addition of macromolecules has a negative impact on translation, and it is reached by the combination of 2.5 $\mathrm{\mu}$M sfGFP RNA with the sfGFP protein produced in the first 0.5 h; (ii) during translation, side products are produced or key energy molecules are consumed, but it is detrimental to translation only in a crowded environment due to decreased diffusion rates. We note that these experimental observations suggest that the ‘toxin’ could be transcription’s mRNA and translation’s sfGFP, leading to macromolecular crowding with a negative effect on translation. Furthermore, we also note that the exact concentration of RNA required to cause the detrimental effects seems to depend on the quality of the prepared RNA, as different RNA batches yielded slightly different critical concentrations for observing this behaviour (Supplementary Fig. S14).

To further interrogate the role of mRNA in translation-induced toxicity, we repeated this experiment in a lysate prepared from a BL21 DE3*ΔlacZ* strain lacking the Star mutation (i.e*.* rne131). The Star mutation, commonly used in crude cell-free expression lysates, truncates the RNaseE gene, resulting in a non-functional RNaseE and functionally increasing mRNA stability. By rescuing full RNaseE expression activity in our lysate, we hoped to further interrogate the effects of mRNA concentration on our hypotheses. Interestingly, we were able to demonstrate a similar decrease in expression at high concentrations of both coding and non-coding reporter mRNA even with RNaseE activity (Supplementary Fig. S15), although—similar to the results shown in Supplementary Fig. S14—the quantitative details of the behaviours observed varied between RNA batches (data not shown). Furthermore, when repression was observed, the onset and extent of repression were both delayed and lessened, respectively. Accordingly, we hypothesize that RNaseE results in a less crowded environment either directly due to decreased RNA concentration or indirectly *via* slower protein expression, further supporting the off-target effects of RNA concentration on translation. In total, these results suggest a complex trade-off between transcription and translation [41], which can result in early cessation of translation.

### Model presents tradeoff in predicting all experimental trends

As mentioned earlier, no sampled model was able to capture all nine criteria representing qualitative experimental trends. However, all of these criteria can be satisfied individually, suggesting that the model structure should be able to capture each of the experimental trends but there is some tradeoff in which criteria to satisfy for each sampled model. Two optimization approaches were used to explore whether all surrogate terms could be satisfied at the same time, namely the parameter estimation mode, which minimizes the sum of the surrogate terms, and the feasibility mode, which treats all criteria as hard constraints and searches for a feasible solution (see more details in Methods). In addition to the parameter estimation mode not returning a set of parameters that satisfied all surrogate terms (Supplementary Fig. S16), all optimization runs in feasibility mode returned infeasible exit flags for satisfying all constraints.

**Figure 7:**
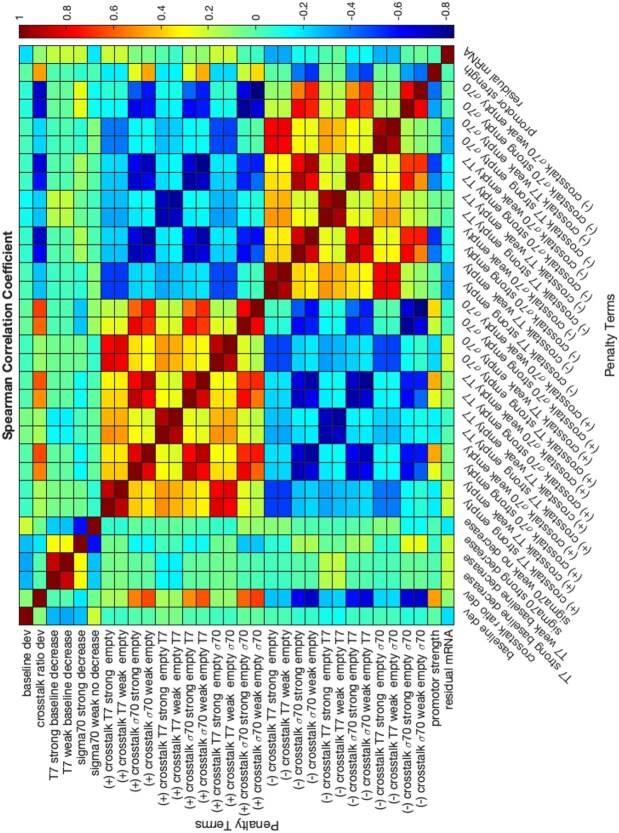
Heatmap for Spearman correlation coefficients between surrogate terms. Surrogate terms are defined in Supplementary Table S2.

To further understand the tradeoff between achieving different qualitative criteria, we calculated the Spearman correlation coefficient between the surrogate terms across all sampled parameter sets and found several pairs that are significantly negatively correlated (Fig. 7). This negative correlation indicates that satisfying one criterion could come at the cost of not satisfying the other. The surrogate terms for decrease in baseline protein expression (with reporter plasmid only) at the highest plasmid concentrations for the three strong promoters are negatively correlated with that for the weak promoter, suggesting difficulty in exhibiting both a decrease in expression for strong promoters and an increase in expression for the weak promoter at higher plasmid concentrations. There is also a negative correlation between positive crosstalk and negative crosstalk surrogate terms in general, suggesting the challenge in capturing both. The correlation of crosstalk ratios with crosstalk ratio deviation and large positive crosstalk ratios is due to the inherent relationship among the terms, as large positive crosstalk requires the existence of positive crosstalk, and violated positive or negative crosstalk surrogate terms are reflected in crosstalk ratio deviation.

## Discussion

In this work, we developed the first kinetic ODE model for lysate-based CFE systems that is able to capture two key experimental trends: a decrease in protein expression at high plasmid concentrations and an increase in reporter protein expression with the addition of orthogonal plasmids in certain concentration regimes. Literature estimates of some parameter values in CFE models (e.g. nuclease concentrations) had to be changed by orders of magnitude to create a model capable of capturing these phenomena. The complex parameterization objective function landscape required the use of surrogate terms reflecting key qualitative trends as an objective function rather than just the minimization of residuals with respect to experimental data. With these changes, the inclusion of multiple new mechanisms allowed for more realistic modelling of plasmid crosstalk phenomena than was previously possible.

This new model suggested that byproducts of translation may be a major contributor to the burden in CFE systems and that these byproducts may directly affect translational potential itself, potentially making it a self-limiting process. Experiments in a translation-only CFE system were able to validate that translation is limited at high levels of mRNA that serve as templates for high translational rates, but also suggested that macromolecular crowding, rather than translation alone, may be an important cause as well.

Analysis on resource utilization *via* tracking of experimentally unmeasurable intermediates suggested that mRNA expressed from the antibiotic resistance gene (or potentially any other DNA template in the reaction, in certain cases) competes for ribonucleases with the reporter mRNA and slows down the degradation of reporter mRNA, leading to higher expression of reporter (positive crosstalk). This occurs when the antibiotic resistance gene is at a higher concentration than the reporter gene or when the reporter gene is transcribed from a weaker promoter. However, when the reporter gene is on a weaker promoter, there could be competition for RNA polymerase between the antibiotic resistance gene and the reporter gene, leading to some offset of the positive crosstalk. The prominent role of the antibiotic resistance gene in plasmid crosstalk was also supported by parameter sensitivity analysis. This suggests the potential importance of careful design of DNA templates for CFE systems and suggests the additional benefit of using linear DNA [58] or antibiotic-resistance-gene-free plasmid [59]. We note, though, that crosstalk does not require the presence of multiple plasmids in cell-free systems: the use of a single plasmid with multiple reporters would still exhibit qualitatively similar crosstalk, though with fine quantitative differences due to a decrease in antibiotic resistance marker expression. Such experiments could just as easily use the model developed here for the presence of multiple plasmids.

While this mathematical model can capture the key experimental trends qualitatively, it is limited in its quantitative accuracy. We have explored avenues to improve our quantitative fitting, including modified reaction mechanisms and expanding parameter bounds, but further model calibration with more experimental data is still necessary to achieve quantitative accuracy. For a large model, it would be beneficial if individual parameters, or the relationship between individual parameters, could be experimentally determined directly and independently, and then treated as fixed values or estimated in a narrower range of values.

Another limitation of this model is its focus on the off-target expression of the specific model protein and RNA, sfGFP and 3WJdB, respectively. The expression of other proteins and oligonucleotides, as would occur in typical real-world applications of cell-free systems, may lead to differences in critical model parameters, therefore limiting the direct translatability of this model’s parameterizations. Specifically, other proteins and oligonucleotides would have varying codon frequency and mRNA secondary structure, which will affect expression rates and NTP consumption as well as the rate of RNA degradation, which would impact the magnitude of plasmid crosstalk. Further investigation of the applicability of a single mathematical model to describe and predict plasmid crosstalk in these scenarios is thus warranted. For example, the various aminoacyl-tRNAs can be tracked as individual species to reflect potential competition for a specific aminoacyl-tRNA, and existing mRNA stability prediction models can be used to inform rates of mRNA degradation based on binding affinity of nucleases to folded transcripts. In addition, there may be uncharacterized expression from cryptic transcription start sites on plasmids that would affect quantitative levels of crosstalk. Until such cryptic transcription is measured experimentally, our model’s accuracy will remain limited by the lack of inclusion of this potential phenomenon.

An accurate mathematical modelling framework for multi-plasmid expression in lysate-based cell-free systems would enable rapid design iterations to express multiple, functionally diverse proteins, peptides, or oligonucleotides for a variety of applications. For instance, when designing a cell-free genetic circuit or multi-enzymatic pathway that requires the production of multiple proteins, the mathematical model could be used to predict the extent of plasmid crosstalk before any significant experimental endeavour is undertaken. If significant off-target, undesirable expression is predicted, additional rounds of model prediction to minimize resource competition through plasmid concentration and strategic template design can be performed, helping to avoid confounding results and wasted experimental efforts. These efforts have the potential to significantly expedite the engineering cycle for biomanufacturing, unlocking new applications.

Many other challenges remain in developing mathematical models to characterize and to predict the behaviour of CFE systems. For example, although there are multiple studies on variability among lysate-based CFE systems depending on different reagents and preparation methods [40, 60], there are currently no commonly accepted mechanistic hypotheses that explain this variability well. Without elucidating these underlying mechanisms, one single model will not be able to capture all variability across lysates. In addition, the endogenous metabolism in lysate-based CFE reactions further complicates modelling efforts, as it affects the levels of molecules, such as ATP and amino acids, required by transcription and translation. Finally, previous studies have sought to connect endogenous metabolism and cell-free protein expression [61, 62], but parameter estimation and model identifiability remain significant challenges in these models.

As the standardization and characterization of CFE systems becomes a community effort, we believe that the mathematical model developed in this manuscript serves as a stepping stone to capturing complex experimental trends and variability among batches. For example, the toxic molecule build-up mechanism can be further expanded to more specific metabolites, enzymes, or cofactors in CFE systems whose concentrations change with time. In addition, the variability in these molecules’ concentrations as a result of differences in lysate processing could potentially be described as different initial conditions for state variables in the mathematical model. These types of digital twins of CFE systems could lead to better experimental design and broader applications.

## Supplementary Material

Supplementary_Material_ysaf011

## Data Availability

The data generated and analysed during the current study are available in the GitHub repository: https://github.com/Yue-Han0/Plasmid-Crosstalk-Modeling.git.

## Material availability

All materials and reagents used in this study were obtained from commercial sources, and the vendors are specified in the Methods section of the manuscript. Plasmid materials are available upon request for academic and non-profit research purposes.
