# Supplementary material for: A mathematical model of cell-free transcription-translation with plasmid crosstalk
Source: Synth Biol (Oxf). 2025 Jun 14;10(1):ysaf011. doi: 10.1093/synbio/ysaf011 (PMC12371409; doi:10.1093/synbio/ysaf011)
Supplement: Supplementary_Material_ysaf011 [file supplementary_material_ysaf011.pdf]

Supplementary Material

# A Mathematical Model of Cell-Free Transcription-Translation with Plasmid Crosstalk

Yue Han[1], Alexandra T. Patterson[1], Fernanda Piorino[1,2], Mark P. Styczynski[1]*

[1] School of Chemical and Biomolecular Engineering, Georgia Institute of Technology, Atlanta, Georgia, United States

[2] Cellular Engineering Group, National Institute of Standards and Technology, Gaithersburg, Maryland, United States

*Corresponding email address: Mark.Styczynski@chbe.gatech.edu

## Supplementary Material Contents

*Experimental Data*

Figure S1. Protein-level plasmid crosstalk

Figure S2. mRNA-level plasmid crosstalk

*Calibration Curves of Fluorescence*

Figure S3. sfGFP calibration curve

Figure S4. 3WJdB RNA aptamer calibration curve

*Model Reaction Network*

*Model Parameter Bounds*

Table S1. Lower and upper bounds for parameters used for sampling

*Surrogate Goals*

Table S2. Surrogate terms for qualitative experimental trends

*Existing Model Performance*

Figure S5. Distribution of surrogate terms in 100 optimization runs

*Alternative RNA Degradation Mechanisms*

Figure S6. Alternative RNA degradation reaction mechanisms

Figure S7. Motivation and results for alternative RNA degradation mechanisms

*Model Calibration*

Figure S8. Deviation and number of satisfied qualitative terms for parameters before and after optimization

Figure S9. Number of times experimental trends are captured

*Additional Supplementary Figures and Tables*

Figure S10. Distribution of surrogate terms for sampled parameter sets that satisfy at least 8 criteria

Figure S11. Simulated transcription-level crosstalk using parameters from selected model

Figure S12. Resource utilization of the selected model at 10 nM reporter plasmid concentration.

Figure S13. Free ribonucleases and ribonucleases occupied by sfGFP and kanR mRNA

Figure S14. RNA prepared from different batches require different concentrations to achieve negative effects at high concentrations

Figure S15. RNA induced translational toxicity occurs in cell-free lysate with RNasE activity

Figure S16. Distribution of surrogate terms for estimated parameter sets

Table S4. DNA sequences used in this paper

## Experimental Data

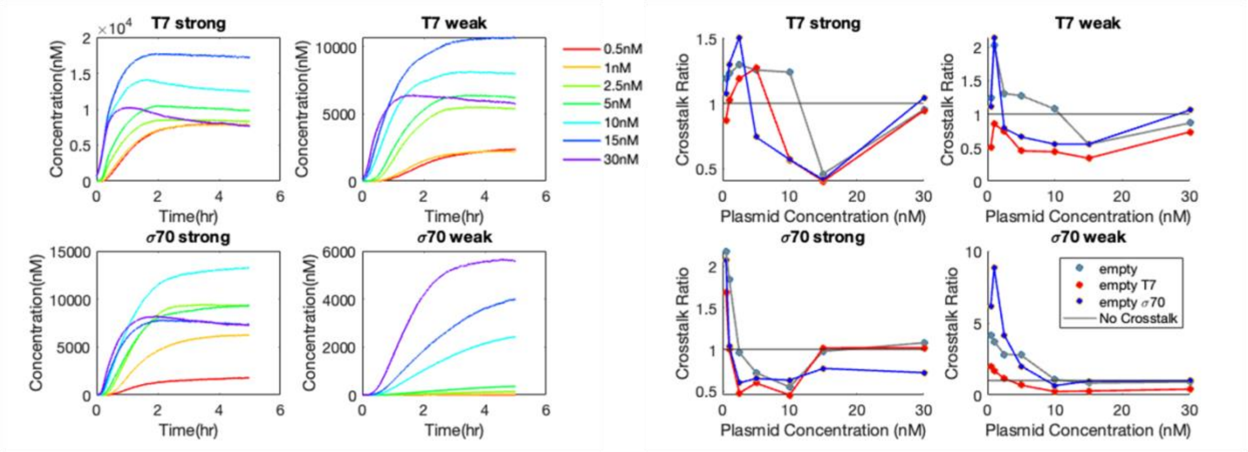


**Figure S1.** Experimental data from plasmid crosstalk reproduced from [1]. (A) sfGFP expression time-course for the baseline case (reporter plasmid only) at 7 reporter plasmid concentrations and 4 promoter strengths. Error bars in plots are removed for the sake of clarity. (B) Crosstalk ratios for sfGFP expression upon addition of empty plasmid vectors as described in the legend. Values larger and smaller than 1 indicate positive crosstalk and negative crosstalk, respectively. Error bars in plots are removed for the sake of clarity.

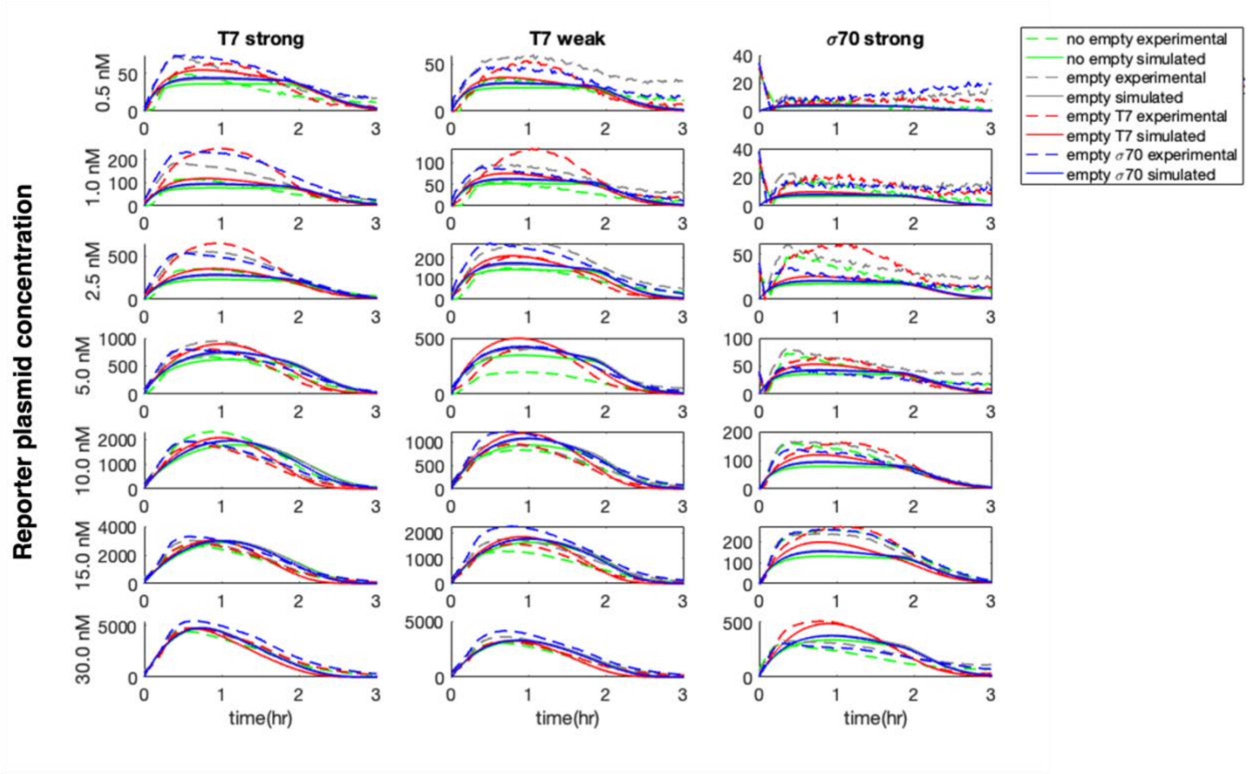


**Figure S2.** Simulated and experimental transcription-level crosstalk using fitted parameters from model calibration only to transcription-level crosstalk data (not the parameterization used for protein expression figures in the main text). Each subplot shows the mRNA concentration over the course of 3 hours for different combinations of plasmids added. Rows correspond to the reporter plasmid concentrations shown on the y-axis and columns correspond to the reporter gene on different promoters. Positive crosstalk occurs when the green curve is below any individual curve and is observed across the subplots.

## Calibration Curves for Fluorescence

To obtain concentration values for the observable species (i.e. 3WJdB RNA aptamer & sfGFP), 7 (including 0 nM) and 8 concentrations of purified sfGFP and *in vitro* transcribed 3WJdB RNA aptamer, respectively, were titrated into cell-free lysates (prepared as described previously [1]). Fluorescence was read at Gain = 50 & 75 and Gain = 70 & 75, respectively. For the 3WJdB RNA aptamer, at Gain = 75, the highest concentration overflows and the lowest concentration did not yield any fluorescence due to the fast degradation of mRNA with native ribonucleases, thus leaving 6 initial concentrations; at Gain = 70, the two lowest initial concentrations did not yield fluorescence output that is differentiable from noise and were thus excluded from the calibration curve. Reactions were run in 10 µL volumes in 384-well small-volume plates (Greiner Bio-One), and fluorescence was measured with a plate reader (Synergy4, BioTek). Excitation and emission wavelengths for sfGFP were 485 and 510 nm, respectively. Excitation and emission wavelengths for 3WJdB were 472 and 507 nm, respectively. Linear regression was performed on sfGFP with the intercept set to 0 (Figure S3). For 3WJdB RNA aptamer, there was a delay between initiation of mRNA degradation—which occurs upon addition of mRNA—and fluorescence reading, so we included a 4-minute time delay in RNA degradation analysis. Each individual fluorescence time course was fitted to a first-order degradation equation ($R^2 > 0.95$, data not shown), which allowed extrapolation of the time course to the actual experimental time zero. Datapoints plotted in Figure S4 are the imputed fluorescence values at the actual time zero, corresponding to the known initial mRNA concentrations.

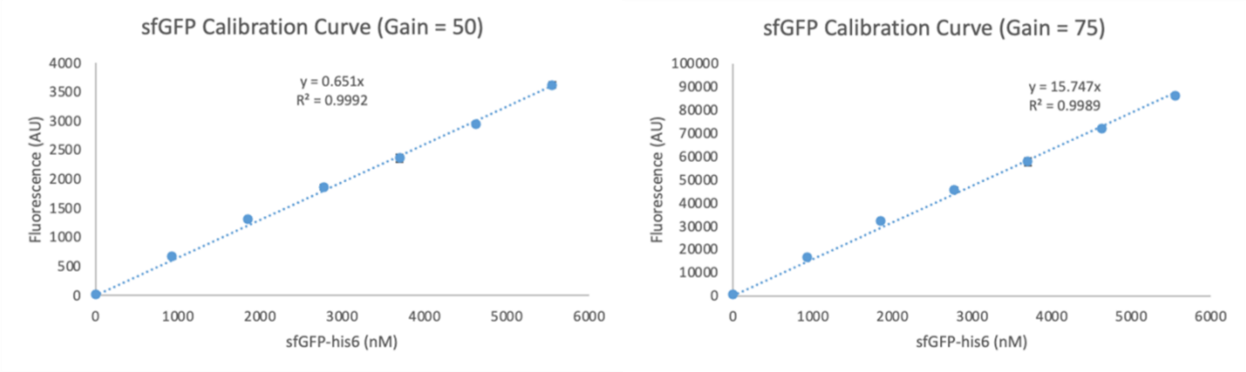


**Figure S3.** sfGFP calibration curve. Data points represent experimental measurements, and the dotted line is a linear regression best fit to the data with a zero intercept. Error bars represent standard deviation from 3 replicate measurements. In most cases they are smaller than the data points.

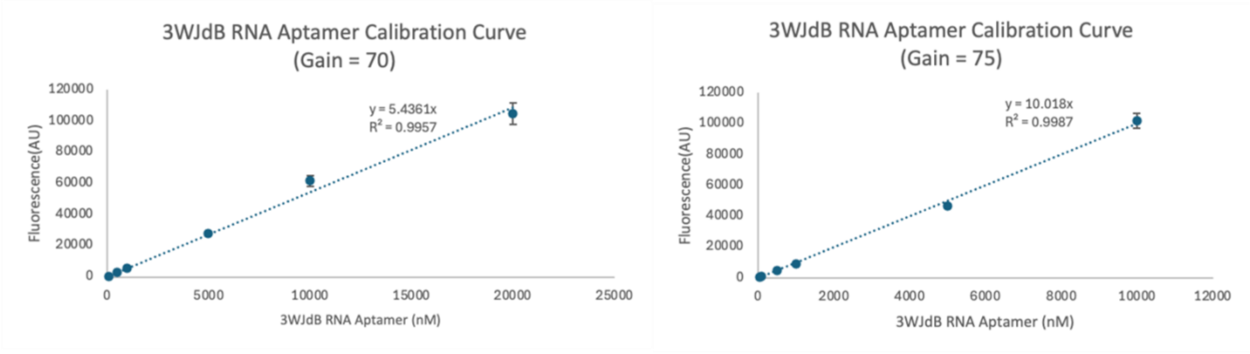


**Figure S4.** 3WJdB RNA aptamer calibration curve. Data points represent imputed fluorescence at the actual time zero from time-course experimental measurements, and the dotted line is a linear regression best fit to the data. Error bars represent standard deviation from 3 replicate measurements. In most cases they are smaller than the data points.

## Model Reaction Network

The reaction network in this model is based on the txtlsim toolbox and reproduced from the supplementary file in [2].

<u>Transcription Reactions:</u>

$$\mathrm{RNAP + DNA} \underset{\mathrm{RNAPbound_R}}{\overset{\mathrm{RNAPbound_F}}{\rightleftharpoons}} \mathrm{RNAP : DNA} \quad (1)$$

$$\mathrm{RNAP : DNA + AGTP} \underset{\mathrm{NTP\ RNAP_{1,R}}}{\overset{\mathrm{NTP\ RNAP_{1,F}}}{\rightleftharpoons}} \mathrm{AGTP : RNAP : DNA} \quad (2)$$

$$\mathrm{RNAP : DNA + CUTP} \underset{\mathrm{NTP\ RNAP_{1,R}}}{\overset{\mathrm{NTP\ RNAP_{1,F}}}{\rightleftharpoons}} \mathrm{CUTP : RNAP : DNA} \quad (3)$$

$$\mathrm{AGTP : RNAP : DNA + CUTP} \underset{\mathrm{NTP\ RNAP_{2,R}}}{\overset{\mathrm{NTP\ RNAP_{2,F}}}{\rightleftharpoons}} \mathrm{CUTP : AGTP : RNAP : DNA} \quad (4)$$

$$\mathrm{CUTP : RNAP : DNA + AGTP} \underset{\mathrm{NTP\ RNAP_{2,R}}}{\overset{\mathrm{NTP\ RNAP_{2,F}}}{\rightleftharpoons}} \mathrm{CUTP : AGTP : RNAP : DNA} \quad (5)$$

$$\mathrm{CUTP : AGTP : RNAP : DNA} \xrightarrow{TX_{elong,glob}/Lm} \mathrm{RNAP : DNA_{term}} + m\,\mathrm{RNA} \quad (6)$$

$$\mathrm{CUTP : AGTP : RNAP : DNA} \xrightarrow{(\frac{Lm}{4}-1)\frac{TX_{elong,glob}}{Lm}} \mathrm{RNAP : DNA} \quad (7)$$

$$\mathrm{RNAP : DNA_{term}} \xrightarrow{RNAPboundTerminationRate} \mathrm{RNAP + DNA} \quad (8)$$

These reactions occur for sfGFP, 3WJdB, kanR, and empty vector in this model. For transcription of genes regulated by T7 promoter family, the RNAP in these reactions is replaced by T7 RNAP. Also, the transcription elongation rate is assumed to be different for $\sigma^{70}$ and T7 polymerases. The parameter $L_m$ refers to the length of transcribed region and is a known value.

Translation Reactions:

$$\mathrm{Ribo} + m\,\mathrm{RNA} \underset{\mathrm{UTR_{gene,R}}}{\overset{\mathrm{UTR_{gene,F}}}{\longleftrightarrow}} \mathrm{Ribo} : m\,\mathrm{RNA} \quad (9)$$
$$\mathrm{Ribo} : m\,\mathrm{RNA} + \mathrm{AA} \underset{\mathrm{AA_R}}{\overset{\mathrm{AA_F}}{\longleftrightarrow}} \mathrm{AA} : \mathrm{Ribo} : m\,\mathrm{RNA} \quad (10)$$
$$\mathrm{AA} : \mathrm{Ribo} : m\,\mathrm{RNA} + \mathrm{AGTP} \underset{\mathrm{AGTP_R}}{\overset{\mathrm{AGTP_F}}{\longleftrightarrow}} \mathrm{AA} : \mathrm{AGTP} : \mathrm{Ribo} : m\,\mathrm{RNA} \quad (11)$$
$$\mathrm{AA} : \mathrm{AGTP} : \mathrm{Ribo} : m\,\mathrm{RNA} \xrightarrow{TL_{elong}/L_p} \mathrm{Ribo} : m\,\mathrm{RNA_{term}} + \mathrm{protein} + \mathrm{AGMP} \quad (12)$$
$$\mathrm{AA} : \mathrm{AGTP} : \mathrm{Ribo} : m\,\mathrm{RNA} \xrightarrow{(Lp-1)TL_{elong}/L_p} \mathrm{Ribo} : m\,\mathrm{RNA} + \mathrm{AGMP} \quad (13)$$
$$\mathrm{Ribo} : m\,\mathrm{RNA_{term}} \xrightarrow{RiboboundTerminationRate} \mathrm{Ribo} + m\,\mathrm{RNA} \quad (14)$$

These reactions occur for sfGFP and kanR in this model. The parameter $L_p$ refers to the length of protein and is a known value.

mRNA degradation reactions:

$$\mathrm{RNase} + m\,\mathrm{RNA} \underset{\mathrm{RNA_{deg,R}}}{\overset{\mathrm{RNA_{deg,F}}}{\longleftrightarrow}} \mathrm{RNase} : m\,\mathrm{RNA} \quad (15)$$
$$\mathrm{RNase} : m\,\mathrm{RNA} \xrightarrow{RNA_{deg,kc}} \mathrm{RNase} \quad (16)$$
$$\mathrm{RNase} + \mathrm{Ribo} : m\,\mathrm{RNA} \underset{\mathrm{RNA_{deg,R}}}{\overset{\mathrm{RNA_{deg,F}}}{\longleftrightarrow}} \mathrm{RNase} : \mathrm{Ribo} : m\,\mathrm{RNA} \quad (17)$$
$$\mathrm{RNase} : \mathrm{Ribo} : m\,\mathrm{RNA} \xrightarrow{RNA_{deg,kc}} \mathrm{RNase} + \mathrm{Ribo} \quad (18)$$
$$\mathrm{RNase} + \mathrm{AA} : \mathrm{Ribo} : m\,\mathrm{RNA} \underset{\mathrm{RNA_{deg,R}}}{\overset{\mathrm{RNA_{deg,F}}}{\longleftrightarrow}} \mathrm{RNase} : \mathrm{AA} : \mathrm{Ribo} : m\,\mathrm{RNA} \quad (19)$$
$$\mathrm{RNase} : \mathrm{AA} : \mathrm{Ribo} : m\,\mathrm{RNA} \xrightarrow{RNA_{deg,kc}} \mathrm{RNase} + \mathrm{AA} + \mathrm{Ribo} \quad (20)$$
$$\mathrm{RNase} + \mathrm{AA} : \mathrm{AGTP} : \mathrm{Ribo} : m\,\mathrm{RNA} \underset{\mathrm{RNA_{deg,R}}}{\overset{\mathrm{RNA_{deg,F}}}{\longleftrightarrow}} \mathrm{RNase} : \mathrm{AA} : \mathrm{AGTP} : \mathrm{Ribo} : m\,\mathrm{RNA} \quad (21)$$
$$\mathrm{RNase} : \mathrm{AA} : \mathrm{AGTP} : \mathrm{Ribo} : m\,\mathrm{RNA} \xrightarrow{RNA_{deg,kc}} \mathrm{RNase} + \mathrm{AGTP} + \mathrm{AA}^{+}\mathrm{Ribo} \quad (22)$$

These reactions occur for all mRNA species (sfGFP, 3WJdB, kanR, empty) in the model. Note that not all reactions are present for 3WJdB and empty mRNA as they do not translate and so have no ribosome/mRNA complexes.

Other reactions:

$$\mathrm{sfGFP} \xrightarrow{PROTsfGFP_{maturation}} \mathrm{sfGFP^*} \quad (23)$$
$$\mathrm{AGMP} \underset{\mathrm{AGTP_{deg,rate}}}{\overset{\mathrm{AGTP_{reg,ON}}}{\longleftrightarrow}} \mathrm{AGTP} \quad (24)$$

These assume that sfGFP has to transform into a matured form before fluorescence is possible [3], and that there is a constant degradation and regeneration of the energy molecules ATP & GTP in the model.

## Model Parameter Bounds

The initial values of model parameters #1-28 are derived from txtlsim toolbox values, and a 0.001x and 1000x value is taken for the lower bound and upper bound for most parameters. The lower and upper bounds for parameters #1 and #2 were initially modified based on [4] to be between [5,100] and [5,30] respectively, and then expanded to 0.01x and 100x (the transcription elongation rate for T7 RNA polymerase is assumed to be faster than that for native *E. coli* RNA polymerase and the upper bound was further increased). The initial values for parameters #18 and #24 (the kinetic rate constants for the unbinding of RNA and ribonucleases) were tuned down approximately three orders of magnitude from the original 3.69e+06 based on optimal fitting from RNA level crosstalk data. The bounds for parameter #27 (the initial ribonuclease concentration) were tuned down to better capture the competition for ribonuclease that contributes to positive plasmid crosstalk, as the initial value was far too high to allow for any competition. The bounds for parameter #28 were derived from an upper value of 2300 nM in [4] and a lower value of 1600 nM in [5] and then expanded two orders of magnitude on each side.

Parameters #29-31 were added to account for the toxic molecule buildup mechanism. As there are no exact literature values, or even direct physical analog, for these parameters, their initial values and bounds were assigned such that the bounds spanned a wide range of magnitudes.

In addition, all forward binding reaction rate constants were fixed at their initial values, since these parameter values can usually be compensated for by the unbinding reaction rate constant, which would give rise to parameter identifiability issues and more challenging parameter estimation. These parameters were fixed during parameter estimation in [2].

**Table S1.** Lower and upper bounds for parameters used for sampling

| Index | Name | Lower Bound | Upper Bound | Parameters for the model shown in Figures 3 & 4 |
|---|---|---|---|---|
| 1 | TX_elong_glob_T7 | 5.00E-02 | 1.00E+04 | 7.13E+02 |
| 2 | TX_elong_glob | 5.00E-02 | 3.00E+03 | 1.44E+01 |
| 3 | TL_elong_glob | 2.38E-02 | 2.38E+04 | 2.20E+03 |
| 4 | AGTPreg_ON | 2.00E-05 | 2.00E+01 | 6.35E-04 |
| 5 | AGTPdeg_rate | 4.87E-08 | 4.87E-02 | 2.91E-04 |
| 6 | TXTL_PT7_RNAPbound_R | 1.00E-04 | 1.00E+04 | 3.01E+01 |
| 7 | TXTL_RNAPBOUND_TERMINATION_RATE | 1.79E-03 | 1.79E+03 | 5.11E-03 |
| 8 | TXTL_NTP_RNAP_1_R | 1.90E-02 | 1.90E+04 | 2.88E+01 |
| 9 | TXTL_NTP_RNAP_2_R | 1.20E+03 | 1.20E+09 | 1.09E+08 |
| 10 | TXTL_RNAdeg_kc | 8.77E-05 | 8.77E+01 | 2.00E-01 |
| 11 | TXTL_PT773_RNAPbound_R | 1.00E-02 | 1.00E+06 | 4.23E+02 |
| 12 | TXTL_PJ23119_RNAPbound_R | 1.00E-02 | 1.00E+06 | 6.11E+00 |
| 13 | TXTL_PROT_sfGFP_MATURATION | 2.31E-06 | 2.31E+00 | 1.32E-03 |
| 14 | TXTL_UTR_GFP_R | 5.92E+01 | 5.92E+07 | 2.16E+04 |
| 15 | TL_AA_R | 5.21E-01 | 5.21E+05 | 6.98E+01 |
| 16 | TL_AGTP_R | 6.03E+02 | 6.03E+08 | 4.70E+07 |
| 17 | TXTL_RIBOBOUND_TERMINATION_RATE | 1.30E-02 | 1.30E+04 | 3.08E-01 |
| 18 | TXTL_RNAdeg_R_sfGFP | 3.69E-01 | 3.69E+06 | 1.88E+00 |
| 19 | TXTL_RNAdeg_kc_sfGFP | 8.77E-06 | 8.77E+00 | 4.73E-05 |
| 20 | TXTL_PJ23105_RNAPbound_R | 1.00E+00 | 1.00E+08 | 6.89E+07 |
| 21 | TXTL_UTR_kanR_R | 5.92E+01 | 5.92E+07 | 1.09E+02 |
| 22 | TXTL_PkanR_RNAPbound_R | 1.00E+00 | 1.00E+08 | 3.60E+02 |
| 23 | TXTL_RNAdeg_R | 1.00E-03 | 3.00E+03 | 1.04E-01 |
| 24 | TXTL_RNAdeg_kc_kanR | 8.77E-06 | 8.77E+00 | 3.39E-05 |
| 25 | RNAP_0 | 1.00E-02 | 1.00E+04 | 1.12E+03 |
| 26 | t7RNAP_0 | 1.00E-02 | 1.00E+04 | 5.53E-02 |
| 27 | RNase_0 | 1.00E-01 | 2.00E+04 | 1.11E+01 |
| 28 | Ribo_0 | 1.60E+01 | 2.30E+05 | 1.92E+02 |
| 29 | tx_capacity_param | 1.00E-03 | 1.00E+05 | 3.87E+01 |
| 30 | k_toxin | 1.00E-04 | 1.00E+04 | 3.14E-02 |
| 31 | toxin_threshold | 3.00E-01 | 1.00E+06 | 5.99E-01 |

## Surrogate Goals

**Table S2.** Surrogate terms for qualitative experimental trends

| Surrogate Term | Description | Goal |
|---|---|---|
| Baseline Deviation | Sum of square deviation between simulated and experimental time-course data for cases with reporter plasmid only | Less than median of sampled values |
| Crosstalk Ratio Deviation | Sum of squares deviation across all concentrations, promoter cases, and plasmid combinations between simulated and experimental crosstalk ratio at t = 3 hours | Less than median of sampled values |
| Baseline Penalty | Increase/decrease of maximum baseline expression from 10 nM ($\sigma^{70}$ strong) or 15 nM (T7 strong & T7 weak & $\sigma^{70}$ weak) to 30 nM of reporter plasmid | Less than -1e-4 |
| Positive & Negative Crosstalk, for each promoter+ | Difference between crosstalk ratio and 1 (no crosstalk) for a given promotor case and plasmid combination* | Less than -0.05 |
| Larger Positive Crosstalk | Difference between the largest crosstalk ratio in $\sigma^{70}$ weak and the largest crosstalk ratio in other promoter cases | Less than -0.05 |
| Residual mRNA | Sum of all mRNA concentrations at the end of time-course | Less than median of sampled values |

+For analysis of the number of criteria satisfied by any individual model parameterization, this criterion was broken into 4 separate criteria corresponding to cases where the reporter gene is controlled by each of the 4 different promoters. Note that for each promoter, there are three sub-criteria corresponding to positive crosstalk for each of the 3 plasmid combinations, and three sub-criteria corresponding to negative crosstalk for each of the 3 plasmid combinations*. For any promoter's criterion to be considered satisfied, the six sub-criteria values must all satisfy the criterion's goal.

*Exception: No criteria for positive crosstalk for T7 weak or empty T7 strong, as positive crosstalk is not always observed in those cases.

## Existing Model Performance

We first explored whether the state-of-the-art modeling toolbox, txtlsim, is able to capture observed plasmid crosstalk trends. Core parameter inference in the txtlsim paper was used as the basis for parameter estimation. We used the initial parameter estimates and the parameter bounds described in [2], as well as the set of assumed fixed parameter values from "Run #3" described in that manuscript's Table 1 (which is used for the plots in that work). 100 optimization runs with initial parameters sampled within parameter bounds were performed. We then calculated values of the surrogate terms for the 100 sets of estimated parameters

(Figure S5). All runs were able to capture negative crosstalk but not positive crosstalk. Also, all runs gave saturation of sfGFP expression levels at high reporter plasmid concentrations rather than a decrease in sfGFP expression, as evidenced by baseline penalty deviations being zero.

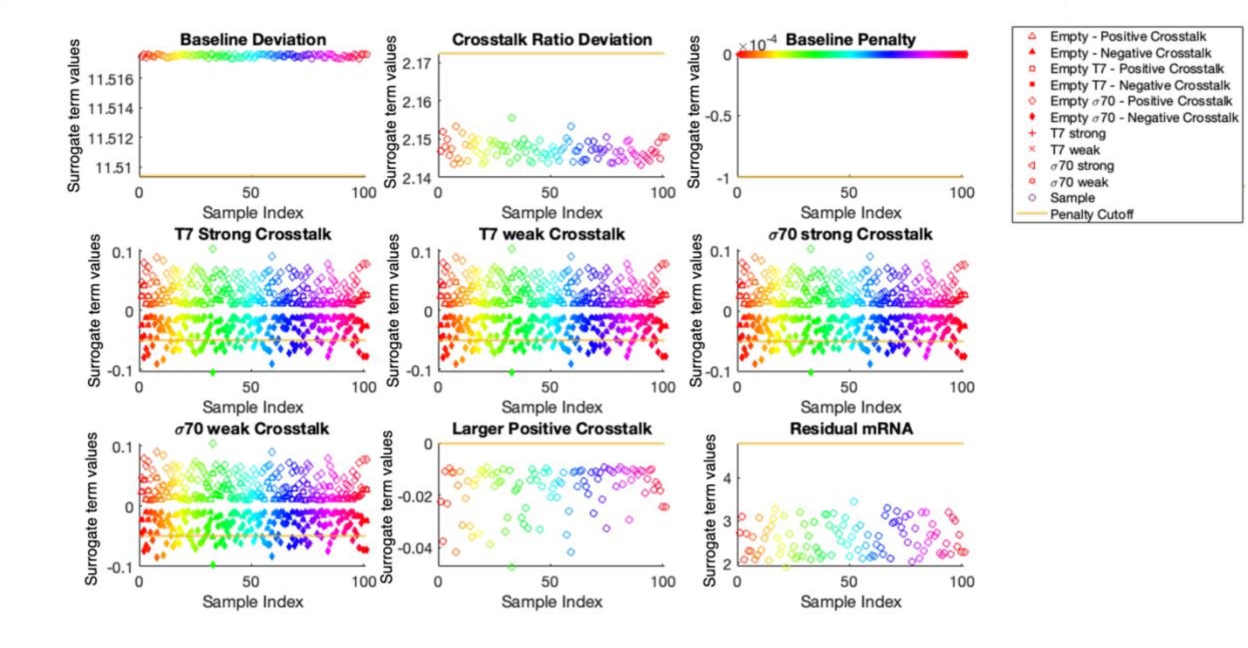


**Figure S5.** Distribution of surrogate term values in 100 optimization runs using existing model. The gold line represents the threshold for each subplot's surrogate term, where markers that fall below the threshold indicate that the surrogate term is satisfied. Baseline deviation penalty, positive crosstalk penalty across all gene regulators, and baseline penalty are not satisfied in any of the 100 optimized models. Note: not all data point markers are used in each subplot. The blank and filled triangle, square, and diamond are only used in T7 strong crosstalk, T7 weak crosstalk, $\sigma^{70}$ strong crosstalk, and $\sigma^{70}$ weak crosstalk. The open circle is used in all other subplots except baseline penalty, where the remaining markers are used.

## Alternative RNA Degradation Mechanisms

An mRNA degradation experiment with 3WJdB was performed in a lysate-based CFE system with different initial mRNA concentrations. We initially fitted degradation with first-order kinetics for extrapolation in the fluorescence calibration curve, which assumes that mRNA degradation is not machinery-limited and is widely accepted as the modeling standard for mRNA degradation. However, upon doing such fitting for individual initial mRNA concentrations, there is a clear decrease in the kinetic rate constant as the mRNA concentration increases, suggesting that degradation machinery may be limiting at high mRNA concentrations. In addition, since ribonucleases in *E.coli*-based cell-free lysates are predominantly non-processive endoribonucleases [6], it is likely that the non-functional mRNA (after first cleavage) still attracts

the endoribonucleases and thus can occupy the degradation machinery in a way that decreases the degradation rate.

To account for this implied competition for ribonucleases, two alternative reaction schemes were proposed for RNA degradation. Mechanism A, shown in Figure S6, explicitly tracks a deactivated mRNA species that is assumed to still have cleavage sites to which ribonuclease (RNase) can bind (we refer to this as the “further degradation” mechanism). Once RNase is bound to a deactivated mRNA and cleavage occurs, the product can be any of the 3 following: (1) 2 pieces of deactivated mRNA with available cleavage sites, (2) 1 piece of deactivated mRNA with available cleavage sites and 1 piece without, or (3) 2 pieces of deactivated mRNA without available cleavage sites. These scenarios are represented by reactions (4)-(6) in Figure S6A. Mechanism B (referred to as the “binding site” mechanism) tracks the number of binding sites instead of mRNA species. Each mRNA species is assumed to contain N sites where ribonucleases can bind, where N is a parameter to be estimated. (We note that with empirical knowledge on mRNA sequences and types of endonucleases in CFE, N should be a known number.) Existing binding sites are divided into two categories: binding sites on functional mRNA, and those on deactivated mRNA. We assume that one endonuclease cut could disable the mRNA’s function, specifically fluorescence in this case. As a result, the mRNA degradation rate is represented by the degradation rate of an occupied site on functional mRNA (i.e. same reaction rate as reaction (3) in Figure S6B).

Models modified with these two mechanisms were used to fit RNA degradation data along with models assuming first-order, Michaelis-Menten, and mass-action kinetics. The two models with the newly proposed mechanisms were able to achieve slightly lower fitting error than the other reaction mechanisms (Figure S7), but this improvement may be due to overfitting given the additional parameters in each of those mechanisms. As a result, we decided not to move forward with these two mechanisms, although we believe that the hidden competition for RNA degradation machinery in cell-free systems by non-functional but not completely degraded mRNA contributes to the non-linear relationships between plasmid concentration and protein expression and is worth further investigation.

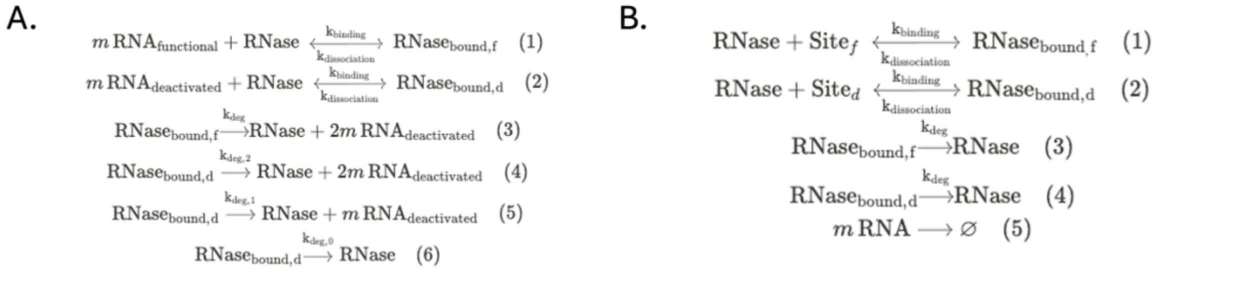

**Figure S6.** Alternative RNA degradation reaction mechanisms. (A) Further degradation mechanism reflecting the presence of additional ribonuclease binding sites in non-functional mRNA degradation products; (B) binding site mechanism that tracks the ribonuclease binding sites as their own species.

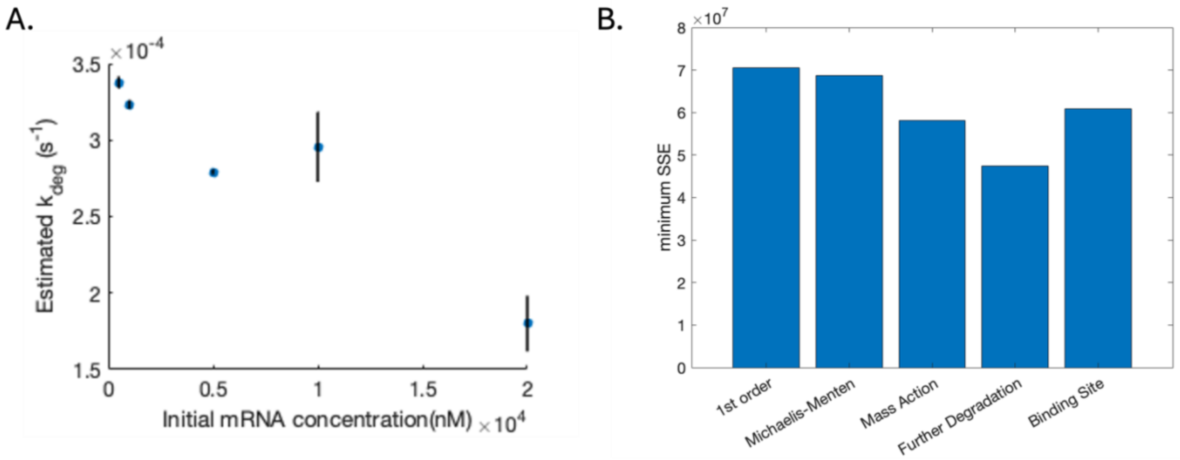


**Figure S7**. Motivation and results for alternative RNA degradation mechanisms. (A) Fitted first-order degradation kinetic constant $k_{deg}$ for various initial mRNA concentrations in CFE reactions. Dots represent average value of 3 technical replicates. At higher initial mRNA concentrations, apparent degradation rates are lower, suggesting that degradation machinery concentration may limit the degradation process. (B) Minimum SSE achieved using alternative RNA degradation mechanisms.

## Model Calibration

Initial efforts focused on fitting the ODE model to experimental data using a conventional objective function describing the sum of squared error between the simulated and experimental data. While the key experimental trends of transition between positive and negative crosstalk and decrease in expression at high plasmid concentrations can be captured in some parameterizations, there is often a tradeoff between optimal objective value and capturing qualitative trends (i.e. the runs where the transition from positive to negative crosstalk are captured have higher deviation from experimental data). Figure S8 shows for 96 sampled parameterizations and 96 optimized parameterizations the number of qualitative trends (described in Table S2 and Figure 1) that are satisfied for each parameterization, along with the corresponding deviation from experimental data (as measured by SSE) of each parameterization. Parameterizations with the lowest deviation do not capture all key experimental trends, and there is no significant correlation between the number of

experimental trends captured and SSE, indicating an inability to capture experimental trends using SSE alone as an objective function.

We first suspected that this was due to the larger numerical values for expression in time courses with high plasmid concentrations being weighted more heavily than for time courses with low plasmid concentrations, where positive crosstalk occurs. To overcome this issue, all time courses were normalized to an endpoint of 1 by dividing each time point's concentration by the final GFP concentration. 96 optimization runs were then performed using the normalized data with the same initial guesses as the original time-courses. However, the data normalization did not improve the model's ability to capture the experimental trends (Figure S9), as can be seen from the similar performances of the original and the normalized experimental data in terms of capturing qualitative experimental trends.

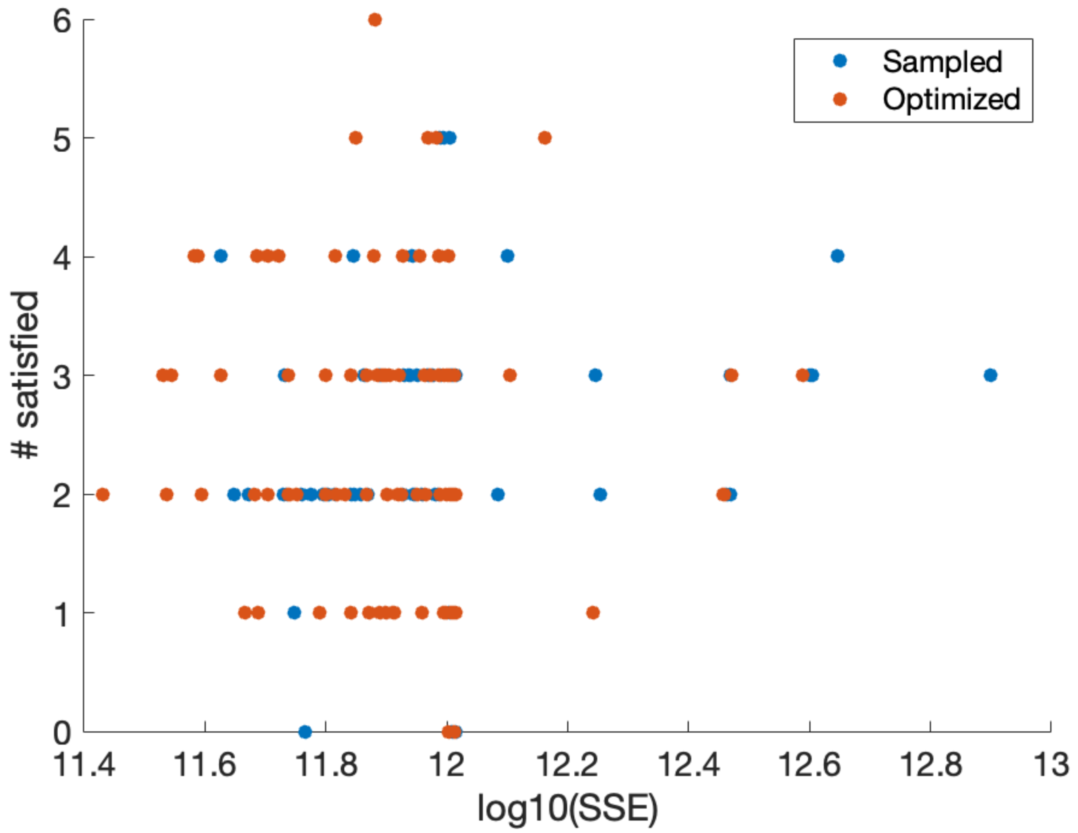


**Figure S8.** Model error and number of satisfied qualitative trends for parameters before and after optimization. Blue dots represent sampled parameters without further optimization, and orange dots represent optimized parameters using blue dots as initial guesses.

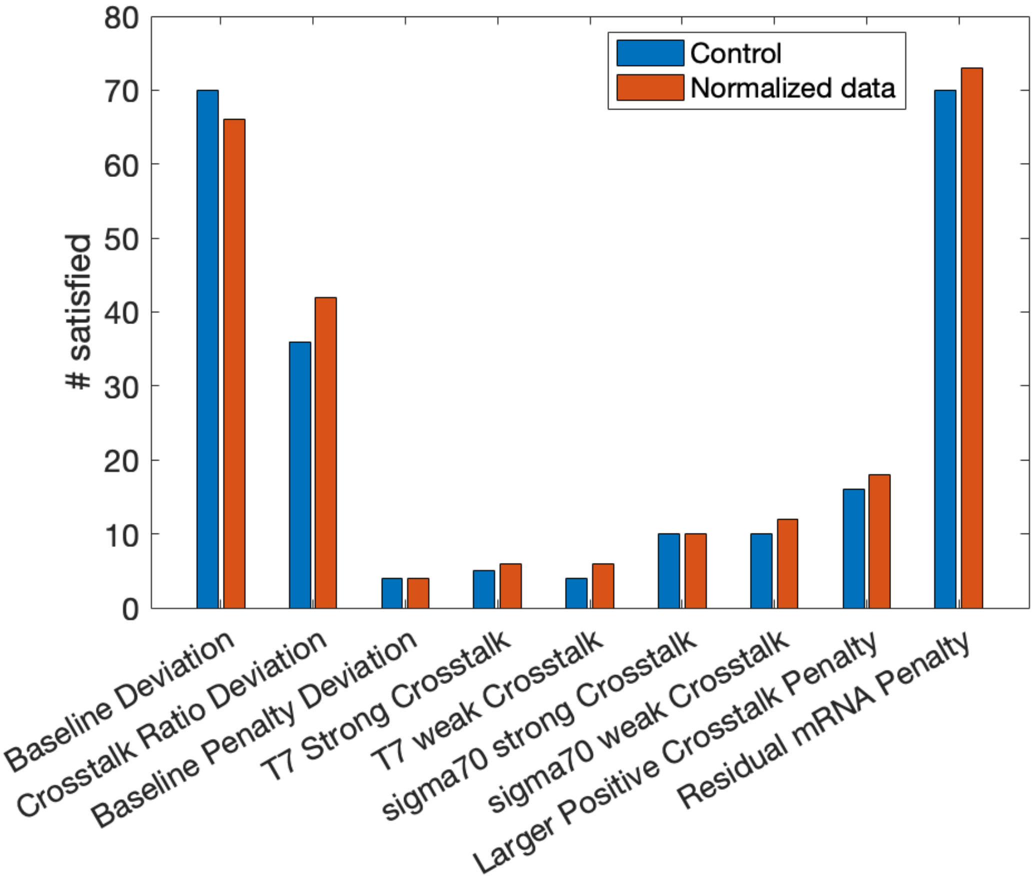


**Figure S9.** Number of times among 96 optimization runs that each experimental trend was captured using SSE as the objective function.

## Additional Supplementary Figures and Tables

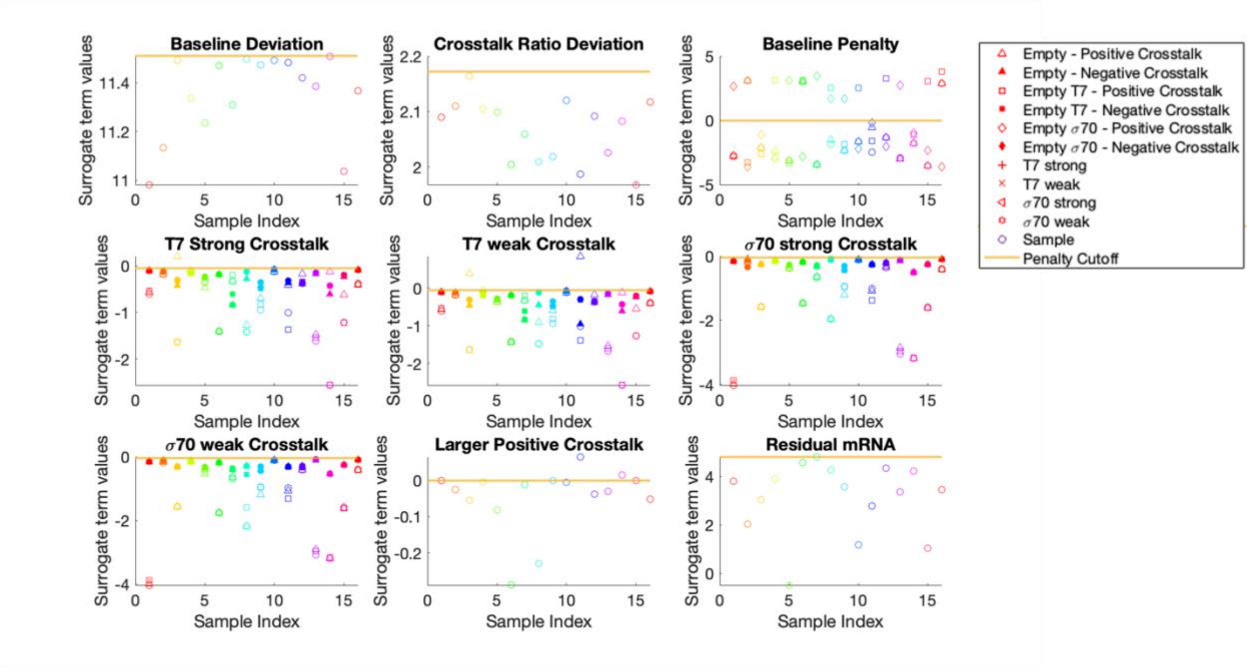


**Figure S10.** Distribution of surrogate term values for sampled parameter sets that satisfy at least 8 criteria. Not all markers are used in each subplot. The blank and filled triangle, square, and diamond are only used in T7 strong crosstalk, T7 weak crosstalk, $\sigma^{70}$ strong crosstalk, and $\sigma^{70}$ weak crosstalk. The open circle is used in all other subplots except baseline penalty, where the remaining markers are used. The gold line represents the threshold for each subplot's surrogate term, where markers that fall below the threshold indicate that the surrogate term is satisfied.

**Table S3.** Number of parameter set samples satisfying a given number of the qualitative trend criteria

| Number of satisfied qualitative trend criteria | Number of parameter set samples |
|---|---|
| **1** | 145780 |
| **2** | 85730 |
| **3** | 25796 |
| **4** | 5538 |
| **5** | 1864 |
| **6** | 734 |
| **7** | 161 |
| **8** | 16 |
| **9** | 0 |

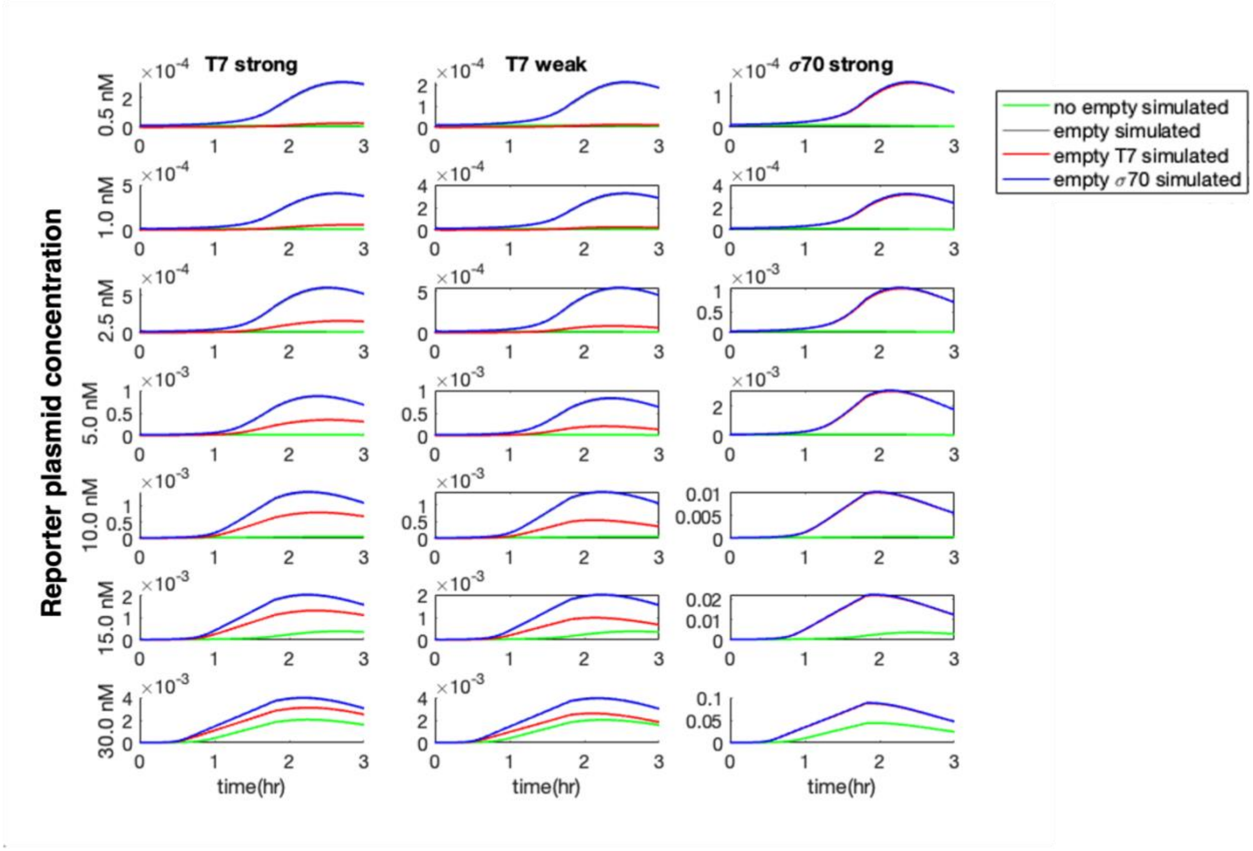


**Figure S11.** Simulated transcription-level crosstalk using parameters from the selected model used for Figure 3. Each subplot shows the mRNA concentration over the course of 3 hours for different combinations of plasmids added. Rows correspond to the reporter plasmid concentrations shown on the y-axis and columns correspond to the reporter gene on different promoters. Positive crosstalk occurs when the green curve is below any individual curve.

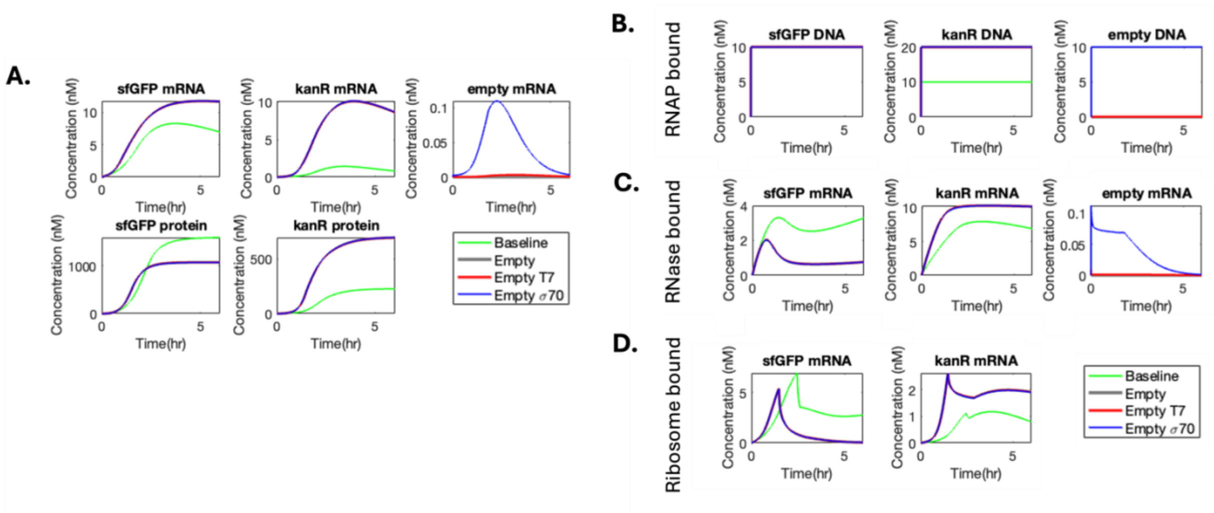


**Figure S12.** Resource utilization of the selected model at 10 nM reporter plasmid concentration. (A) The concentration profile of mRNA (excluding those bound to ribonucleases) produced from the sfGFP, kanR, and empty vector genes and of kanR and sfGFP protein, over the simulated course of 6 hours. (B) Concentration of native RNA polymerase bound with each of the sfGFP, kanR, and empty vector gene over time. (C) Concentration of ribonucleases bound with each of sfGFP, kanR, and empty mRNA over time. (D) Concentration of ribosomes bound with sfGFP and kanR mRNA over time.

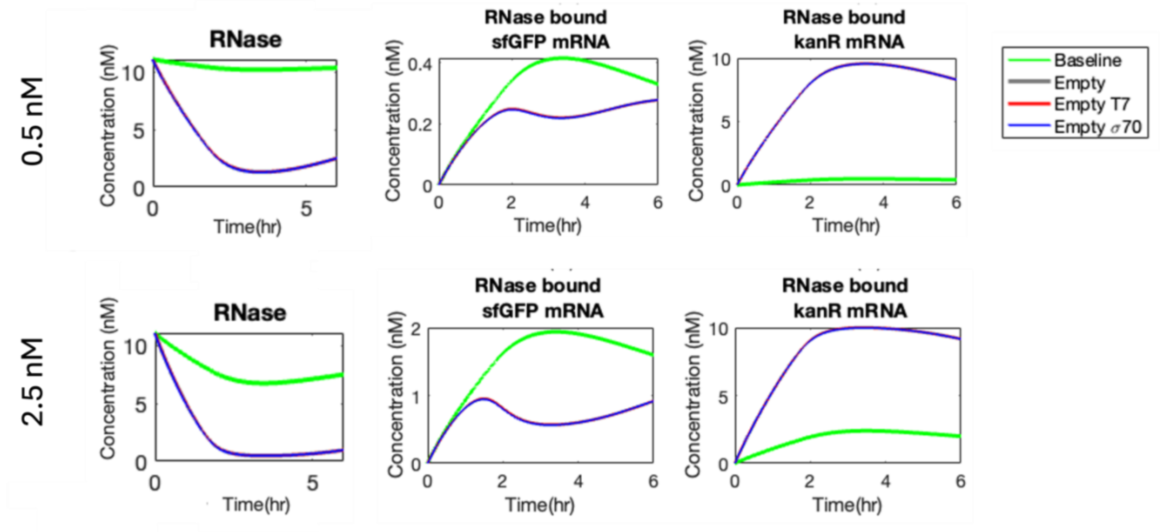


**Figure S13.** Free ribonucleases and ribonucleases occupied by sfGFP and kanR mRNA at 0.5 & 2.5 nM reporter plasmid concentration over the simulated time course of 6 hours. With additional plasmids expressing kanR, kanR bound ribonuclease concentration increases at a higher rate at 2.5 nM than 0.5 nM, causing fewer available ribonucleases for sfGFP mRNA to bind to. As a result, a smaller proportion of sfGFP mRNA of all produced mRNA is bound to ribonucleases, causing the larger positive crosstalk at 2.5 nM.

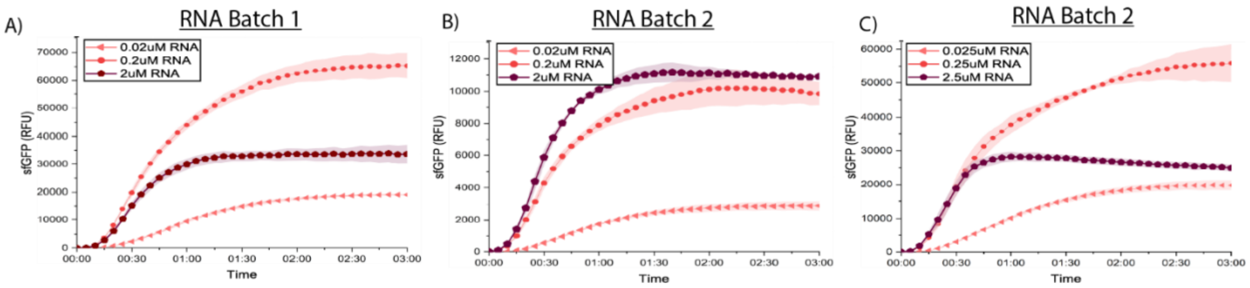


**Figure S14**. RNA prepared from different batches require different concentrations to achieve negative effects at high concentrations. *In vitro* synthesized sfGFP RNAs prepared on different days were added to BL21DE3StarΔLacZ CFE systems at 0.025, 0.25, and 2.5 µM. In RNA Batch 1 (A), there is a clear decrease in sfGFP expression from 0.2 µM (dark pink) to 2 µM (purple) of sfGFP RNA. However, 2.5µM of RNA, as opposed to 2µM, was required to cause negative effects for RNA Batch 2 (B & C). These differences can likely be attributed to differences in RNA quality. However, the phenotype of translational toxicity can be observed in all RNA batches.

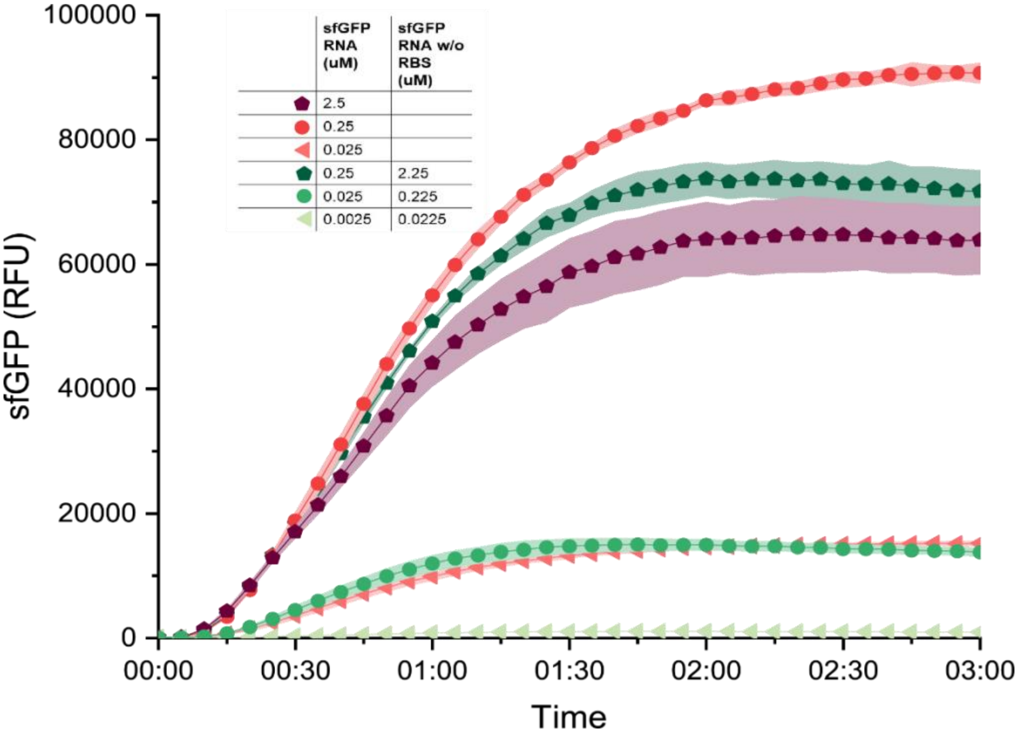


**Figure S15.** RNA-induced translational toxicity occurs in cell-free lysate with RNaseE activity. *In vitro* synthesized sfGFP RNAs with and without RBS were added to BL21DE3ΔLacZ CFE systems at 0.0025, 0.025, 0.25, and 2.5 µM. Even with full RNaseE activity, high levels of RNA (with and without RBS) were able to induce translational toxicity.

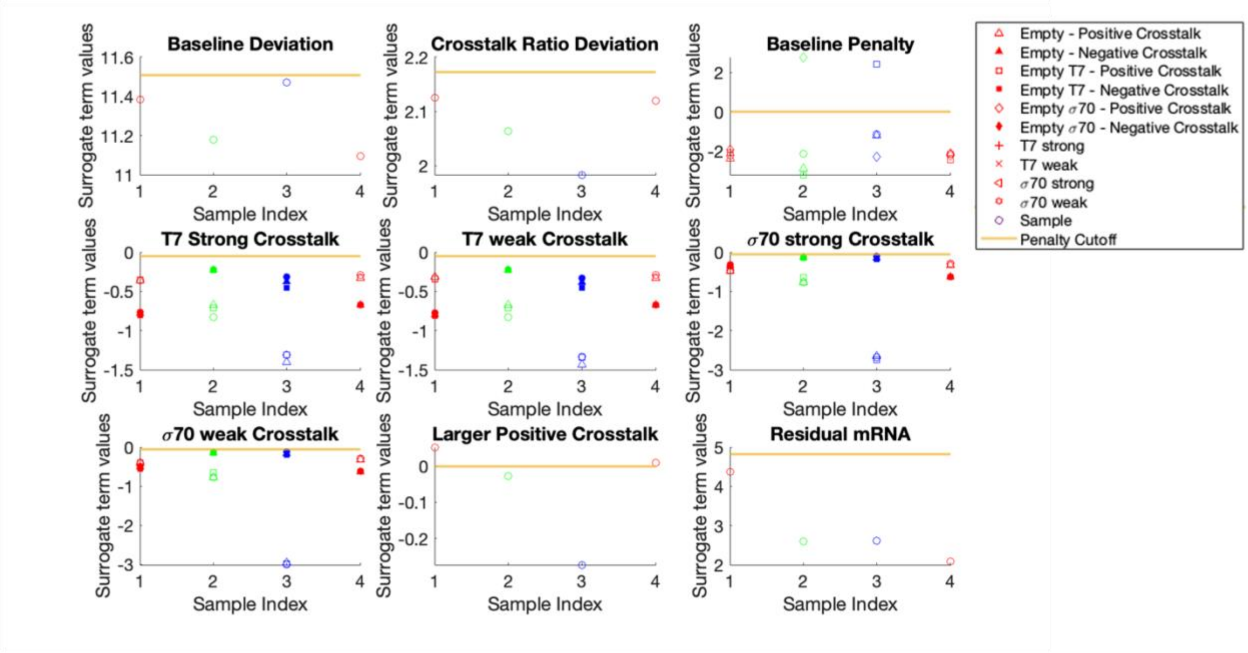


**Figure S16.** Distribution of surrogate terms for estimated parameter sets, using sum of surrogate terms as objective function, where at least 8 criteria is satisfied. Not all markers are used in each subplot. The blank and filled triangle, square, and diamond are only used in T7 strong crosstalk, T7 weak crosstalk, $\sigma^{70}$ strong crosstalk, and $\sigma^{70}$ weak crosstalk. The open circle is used in all other subplots except baseline penalty, where the remaining markers are used. The gold line represents the threshold for each subplot's surrogate term, where markers that fall below the threshold indicate that the surrogate term is satisfied.

**Table S4.** Description of DNA sequences in this paper.

The PT7,strong sfGFP plasmid can be purchased from Addgene as the pJL1 plasmid, #69496. The PT7,strong sfGFP, no RBS linear expression template uses the pJL1 plasmid as a backbone.

<table>
<tr><td>$P_{T7,strong\ sfGFP,\ no\ RBS}$</td><td>Linear DNA template used for in vitro transcription encoding sfGFP expression under $P_{T7,strong}$ without an RBS to ensure no translation</td></tr>
<tr><td>Forward Primer</td><td>gaaattaatacgactcactatagggaga_AGCAAAGGTGAAGAACTGTTTACC</td></tr>
<tr><td>Reverse Primer</td><td>GATAACCTTATTTTTGACGAGGGG</td></tr>
<tr><td colspan="2">$P_{T7,strong}$ sfGFP Terminator Kanamycin resistance cassette ColE1 origin<br><br>gaaattaatacgactcactatagggagaAGCAAAGGTGAAGAACTGTTTACCGGCGTTGTGCCGATTCTGGTGG<br>AACTGGATGGCGATGTGAACGGTCACAAATTCAGCGTGCGTGGTGAAGGTGAAGGCGATGCCACGATT<br>GGCAAACTGACGCTGAAATTTATCTGCACCACCGGCAAACTGCCGGTGCCGTGGCCGACGCTGGTGAC<br>CACCCTGACCTATGGCGTTCAGTGTTTTAGTCGCTATCCGGATCACATGAAACGTCACGATTTCTTTAAAT</td></tr>
</table>

CTGCAATGCCGGAAGGCTATGTGCAGGAACGTACGATTAGCTTTAAAGATGATGGCAAATATAAAACGC
GCGCCGTTGTGAAATTTGAAGGCGATACCCTGGTGAACCGCATTGAACTGAAAGGCACGGATTTTAAA
GAAGATGGCAATATCCTGGGCCATAAACTGGAATACAACTTTAATAGCCATAATGTTTATATTACGGCGGA
TAAACAGAAAAATGGCATCAAAGCGAATTTTACCGTTCGCCATAACGTTGAAGATGGCAGTGTGCAGCT
GGCAGATCATTATCAGCAGAATACCCCGATTGGTGATGGTCCGGTGCTGCTGCCGGATAATCATTATCTG
AGCACGCAGACCGTTCTGTCTAAAGATCCGAACGAAAAAGGCACGCGGGACCACATGGTTCTGCACGA
ATATGTGAATGCGGCAGGTATTACGTGGAGCCATCCGCAGTTCGAAAAATAAgtcgaccggctgctaacaaagc
ccgaaaggaagctgagttggctgctgccaccgctgagcaataactagcataaccccttggggcctctaaacgggtcttgaggggttttt
gctgaaagccaattctgattagaaaaactcatcgagcatcaaatgaaactgcaatttattcatatcaggattatcaataccatatttttga
aaaagccgtttctgtaatgaaggagaaaactcaccgaggcagttccataggatggcaagatcctggtatcggtctgcgattccgactcg
tccaacatcaatacaacctattaatttcccctcgtcaaaaataaggttatc

| $P_{T7,strong}$ sfGFP | Linear DNA template used for *in vitro* transcription encoding sfGFP expression under $P_{T7,strong}$ |
|---|---|
| Forward Primer | cgcgaaattaatacgactcactatagg |
| Reverse Primer | GATAACCTTATTTTTGACGAGGGG |

$P_{T7,strong}$ Stability hairpin RBS sfGFP Terminator Kanamycin resistance cassette ColE1 origin

cgcgaaattaatacgactcactatagggagaccacaacggtttccctctagaaataattttgtttaactttaagaaggagatatacatAT
GAGCAAAGGTGAAGAACTGTTTACCGGCGTTGTGCCGATTCTGGTGGAACTGGATGGCGATGTGAACG
GTCACAAATTCAGCGTGCGTGGTGAAGGTGAAGGCGATGCCACGATTGGCAAACTGACGCTGAAATTT
ATCTGCACCACCGGCAAACTGCCGGTGCCGTGGCCGACGCTGGTGACCACCCTGACCTATGGCGTTCA
GTGTTTTAGTCGCTATCCGGATCACATGAAACGTCACGATTTCTTTAAATCTGCAATGCCGGAAGGCTAT
GTGCAGGAACGTACGATTAGCTTTAAAGATGATGGCAAATATAAAACGCGCGCCGTTGTGAAATTTGAA
GGCGATACCCTGGTGAACCGCATTGAACTGAAAGGCACGGATTTTAAAGAAGATGGCAATATCCTGGG
CCATAAACTGGAATACAACTTTAATAGCCATAATGTTTATATTACGGCGGATAAACAGAAAAATGGCATCA
AAGCGAATTTTACCGTTCGCCATAACGTTGAAGATGGCAGTGTGCAGCTGGCAGATCATTATCAGCAGA
ATACCCCGATTGGTGATGGTCCGGTGCTGCTGCCGGATAATCATTATCTGAGCACGCAGACCGTTCTGTC
TAAAGATCCGAACGAAAAAGGCACGCGGGACCACATGGTTCTGCACGAATATGTGAATGCGGCAGGTA
TTACGTGGAGCCATCCGCAGTTCGAAAAATAAgtcgaccggctgctaacaaagcccgaaaggaagctgagttggctgct
gccaccgctgagcaataactagcataaccccttggggcctctaaacgggtcttgaggggttttttgctgaaagccaattctgattagaaa
aactcatcgagcatcaaatgaaactgcaatttattcatatcaggattatcaataccatatttttgaaaaagccgtttctgtaatgaaggag
aaaactcaccgaggcagttccataggatggcaagatcctggtatcggtctgcgattccgactcgtccaacatcaatacaacctattaatt
tcccctcgtcaaaaataaggttatc

## Supplementary References


1. Piorino F, Patterson AT, Han Y, Styczynski MP: **Plasmid Crosstalk in Cell-Free Expression Systems**. *ACS Synth Biol* 2023, **12**(10):2843-2856.
2. Singhal V, Tuza ZA, Sun ZZ, Murray RM: **A MATLAB toolbox for modeling genetic circuits in cell-free systems**. *Synth Biol (Oxf)* 2021, **6**(1):ysab007.

3. Balleza E, Kim JM, Cluzel P: **Systematic characterization of maturation time of fluorescent proteins in living cells**. *Nature Methods* 2017, **15**(1):47-51.
4. Garamella J, Marshall R, Rustad M, Noireaux V: **The All E. coli TX-TL Toolbox 2.0: A Platform for Cell-Free Synthetic Biology**. *ACS Synth Biol* 2016, **5**(4):344-355.
5. Vilkhovoy M, Horvath N, Shih CH, Wayman JA, Calhoun K, Swartz J, Varner JD: **Sequence Specific Modeling of E. coli Cell-Free Protein Synthesis**. *ACS Synth Biol* 2018, **7**(8):1844-1857.
6. Bechhofer DH, Deutscher MP: **Bacterial ribonucleases and their roles in RNA metabolism**. *Crit Rev Biochem Mol Biol* 2019, **54**(3):242-300.
